# The interaction of resource use and gene flow on the phenotypic divergence of benthic and pelagic morphs of Icelandic Arctic charr (*Salvelinus alpinus*)

**DOI:** 10.1002/ece3.7563

**Published:** 2021-05-02

**Authors:** Matthew K. Brachmann, Kevin Parsons, Skúli Skúlason, Moira M. Ferguson

**Affiliations:** ^1^ Department of Integrative Biology University of Guelph Guelph ON Canada; ^2^ Institute of Biodiversity, Animal Health and Comparative Medicine School of Life Science University of Glasgow Glasgow UK; ^3^ Department of Aquaculture and Fish Biology Hólar University Saudárkrókur Iceland; ^4^ Icelandic Museum of Natural History Reykjavík Iceland

**Keywords:** adaptive divergence, ecological speciation, habitat divergence, morphology, natural selection, phenotype–environment correlation, trophic polymorphism

## Abstract

Conceptual models of adaptive divergence and ecological speciation in sympatry predict differential resource use, phenotype–environment correlations, and reduced gene flow among diverging phenotypes. While these predictions have been assessed in past studies, connections among them have rarely been assessed collectively. We examined relationships among phenotypic, ecological, and genetic variation in Arctic charr (*Salvelinus alpinus*) from six Icelandic localities that have undergone varying degrees of divergence into sympatric benthic and pelagic morphs. We characterized morphological variation with geometric morphometrics, tested for differential resource use between morphs using stable isotopes, and inferred the amount of gene flow from single nucleotide polymorphisms. Analysis of stable isotopic signatures indicated that sympatric morphs showed similar difference in resource use across populations, likely arising from the common utilization of niche space within each population. Carbon isotopic signature was also a significant predictor of individual variation in body shape and size, suggesting that variation in benthic and pelagic resource use is associated with phenotypic variation. The estimated percentage of hybrids between sympatric morphs varied across populations (from 0% to 15.6%) but the majority of fish had genotypes (ancestry coefficients) characteristic of pure morphs. Despite evidence of reduced gene flow between sympatric morphs, we did not detect the expected negative relationship between divergence in resource use and gene flow. Three lakes showed the expected pattern, but morphs in the fourth showed no detectable hybridization and had relatively low differences in resource use between them. This coupled with the finding that resource use and genetic differentiation had differential effects on body shape variation across populations suggests that reproductive isolation maintains phenotypic divergence between benthic and pelagic morphs when the effects of resource use are relatively low. Our ability to assess relationships between phenotype, ecology, and genetics deepens our understanding of the processes underlying adaptive divergence in sympatry.

## Graphical abstract

Our study assesses the contribution of differential resource use, phenotype–environment correlations, and reductions in gene flow to adaptive divergence of benthic–pelagic morphs of Icelandic Arctic charr in sympatry. We integrate a variety of analytical approaches to test our hypotheses using geometric morphometrics, stable isotopic signatures, and single nucleotide polymorphisms. Resource use appears to be the agent of selection driving phenotypic divergence along a benthic–pelagic axis and reproductive isolation may maintain this divergence when the intensity of divergent selection is weak.

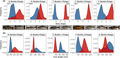

## 1 INTRODUCTION

Understanding the processes by which adaptive divergence and barriers to gene flow evolve between subpopulations remains a challenging problem in evolutionary biology. Conceptual models have posited that adaptive divergence and ecological speciation can occur as the result of ecologically based divergent selection between environments (Rundle & Nosil, 2005; Schluter, 2000, 2009). Accordingly, divergence is initiated by ecological opportunity due to the availability of niche space (Burress & Tan, 2017; Losos, 2010; Wellborn & Langerhans, 2014) and the utilization of those niches by different phenotypes (ecological release) (Burress & Tan, 2017; Des Roches et al., 2011; Losos, 2010; Parent & Crespi, 2009; Wellborn & Langerhans, 2014). Ecological opportunity and release may then result in the evolution of phenotypically discrete subpopulations that utilize different resources in alternative habitats (Häkli et al., 2018; Jarvis et al., 2020; Yoder et al., 2010) as the result of divergent selection. Divergent selection may in turn lead to the build‐up of reproductive isolation associated with the ecology of each habitat (Crispo et al., 2006; Ferris & Willis, 2018; Funk et al., 2006). The rapid build‐up of reproductive isolating mechanisms is expected to result in strong genetic population structuring due to reduced gene flow between diverging subpopulations. This sequence of events may eventually lead to speciation (Muschick et al., 2020; Nosil et al., 2009; Rundle & Nosil, 2005; Schumer et al., 2017), although the frequency of this outcome is still debated (Bird et al., 2012; Coyne & Orr, 2004; Mallet et al., 2009). Determining how the different elements interact, and perhaps reinforce each other, to promote adaptive divergence and speciation will provide a better understanding how phenotypic variation is partitioned in the presence of gene flow.

In cases of adaptive divergence, niche availability is often indirectly inferred by the observation that phenotypically discrete subpopulations or morphs utilize different ecological resources in response to habitat heterogeneity (Wellborn & Langerhans, 2014). Novel environments such as those that emerge in lakes after glacial retreat may provide multiple open niche spaces (ecological opportunities) with available trophic resources utilized by recolonizing individuals (Ribeiro et al., 2018; Robinson & Wilson, 1994; Siwertsson et al., 2010). Colonization of localities with comparable resources and ecological opportunities should result in similar (i.e., parallel) patterns of habitat utilization and phenotypic variation (Wellborn & Langerhans, 2014). Parallel patterns of differential resource specialization among subpopulations in fishes of many northern lakes are often associated with similar patterns of phenotypic differentiation (Berchtold et al., 2015; Franklin et al., 2018; Häkli et al., 2018; Siwertsson, Knudsen, Præbel, et al., 2013). However, the availability of resources (amount of open niche space) will vary due to local or population‐specific factors causing variation in resource use across populations (Arlettaz et al., 2017; Atuo & O'Connell, 2017; Jónsson & Skúlason, 2000; Parent & Crespi, 2009). This in turn can lead to phenotypic outcomes where populations occupy different positions along a continuum of phenotypic divergence (Bolnick et al., 2018; Manousaki et al., 2013; Siwertsson et al., 2013). Recent studies of relatively young adaptive radiations indicate that the extent of phenotypic divergence and resource specialization can be predicted by ecosystem size (Doenz et al., 2019; Jacobs et al., 2020; Lucek et al., 2016). However, uncertainty remains as to how variation in resource use affects phenotypic outcomes and the development of reproductive isolation. The ability to generalize such relationships across populations may allow for better predictions of the nature and outcomes of adaptive divergence.

The presence of phenotype–environment correlations such as those between body shape and resource use might reflect the existence of performance trade‐offs between habitats (Arnegard et al., 2014; Camacho & Hendry, 2020; Harrod et al., 2010; MacColl, 2011; Stroud & Losos, 2016; Wellborn & Langerhans, 2014). Performance trade‐offs often occur along environmental gradients where diverging subpopulations are specialized to utilize a particular habitat‐specific resource (Edelaar et al., 2008; Schluter, 2000; Widmer et al., 2020). Body shape‐dependent resource use arises as morphologically intermediate individuals are expected to compete poorly for an alternative set of discrete resources due to physical constraints leading to reduced fitness. However, phenotypic differences can also arise and persist in sympatry through phenotypic plasticity or matching habitat choice of phenotypes (Edelaar et al., 2017; Nicolaus & Edelaar, 2018). These explanations, plus observations from studies that focus on variation at the interindividual level, are not necessarily consistent with the existence of performance trade‐offs (Franklin et al., 2018) and indicate that we do not fully understand how adaptation to alternative habitats proceeds in natural populations.

While resource specialization in response to habitat heterogeneity can promote adaptive divergence, a reduction in gene flow between habitats is required for progress toward ecological speciation or the maintenance of genetically divergent phenotypes (Kulmuni et al., 2020; Öhlund et al., 2020; Rundle & Nosil, 2005; Smadja & Butlin, 2011). Rapid shifts in habitat divergence can strongly increase the degree of reproductive isolation between diverging subpopulations (Muschick et al., 2020). Reproductive isolation can arise through an increase in assortative mating if diverging subpopulations are exposed to environmental differences that result in varied timing and location of reproduction thus decreasing the likelihood of gene flow (DeRito et al., 2010; Devaux & Lande, 2009; Doenz et al., 2018; Ferris & Willis, 2018; Skúlason et al., 1989). The observation that phenotypically diverged subpopulations are genetically differentiated suggests reduced gene flow (Doenz et al., 2019; Le Moan et al., 2016; Seneviratne et al., 2016; Whitney et al., 2018) but does not necessarily indicate that habitat divergence is the underlying cause (Hendry & Taylor, 2004; Räsänen & Hendry, 2008). Studies that link habitat divergence with gene flow (Bittner et al., 2010; Seneviratne et al., 2016; Whitney et al., 2018) have provided compelling evidence that habitat divergence can indirectly lead to assortative mating and reduced gene flow (Hendry & Taylor, 2004; Perini et al., 2020; Räsänen & Hendry, 2008; Westram et al., 2018; Whitney et al., 2018). However, assessing the relationships between habitat divergence and gene flow does not necessarily identify the exact reproductive isolating mechanism operating within a population (Kulmuni et al., 2020). Moreover, speciation may not always occur due to the interactions of selection and assortative mating (Servedio & Hermisson, 2020).

In order to address some of the above knowledge gaps, we take advantage of a suitable model system. Icelandic Arctic charr (*Salvelinus alpinus*) provide a powerful opportunity to study the interacting ecological and evolutionary mechanisms that underlie adaptive divergence in the face of gene flow. Arctic charr have diverged into discrete resource‐based morphs after recolonization of postglacial lakes within the last 10,000 years (Klemetsen, 2010; Snorrason & Skúlason, 2004). Benthic morphs typically have a subterminal mouth and a deep and stocky body, while pelagic morphs have a terminal mouth and fusiform (torpedo) body shape (Gíslason et al., 1999; Jónsson & Skúlason, 2000; Snorrason et al., 1994). Benthic morphs feed on benthic invertebrates in the benthic or littoral zones while pelagic morphs utilize zooplankton or fish resources in the pelagic zone of the lake (Jónsson & Skúlason, 2000; Knudsen et al., ,^2014^, ^2016^; Malmquist et al., 1992). This pattern of phenotypic divergence along a benthic–pelagic ecological axis persists in different lakes despite potential variation in evolutionary histories ranging from postglacial divergence in sympatry to preglacial divergence with postglacial secondary contact, highlighting the prominent role of natural selection in promoting divergence (Jacobs et al., 2020). Associations between resource use and morphology have been observed in Arctic charr (Adams et al., 2003; Doenz et al., 2019; Franklin et al., 2018; Jónsson & Skúlason, 2000; Kristjánsson et al., 2011), and resource use is thought to be the agent of selection driving phenotypic divergence between benthic and pelagic morphs (Skúlason & Smith, 1995; Smith & Skúlason, 1996). These ideas combined with known genetic differentiation between sympatric benthic and pelagic morphs (Arbour et al., 2011; Gíslason et al., 1999; Guðbrandsson et al., 2019; Kapralova et al., 2011) lead to the hypothesis that ecologically based divergent selection acting on phenotypic variation causes habitat divergence, reduced gene flow between sympatric benthic and pelagic morphs, and the development of reproductive isolation. Reproductive isolation could arise through assortative mating because of known variation in timing, location, and depth of spawning between morphs due to the ecological characteristics of each habitat (Sandlund et al., 1992; Skúlason, Snorrason, et al., 1989) as well as postzygotic isolation (Kapralova, 2014). Regardless of the inferred linkages among morphology, resource use, and genetic differentiation in this species, studies directly assessing interactions among these factors are rare (but see Doenz et al., 2019; Jacobs et al., 2020).

We examined relationships among variation in body shape and size, resource use, and gene flow in benthic and pelagic morphs of Icelandic Arctic charr that vary in phenotypic divergence. We assessed several predictions of the hypothesis that ecologically based divergent selection acting on phenotypic variation has led to adaptive divergence and reduced gene flow between sympatric benthic and pelagic morphs. First, we determined if sympatric benthic and pelagic morphs differ in resource use as measured by stable isotopic signatures and if the pattern is similar across lakes. Common patterns of resource use across lakes would suggest that morphs are utilizing similar niche space along an ecological benthic and pelagic axis. Second, we determined if resource use, the assumed agent of selection, is a significant predictor of body shape and size, the presumed targets of selection. The detection of such phenotype–environment relationships could reflect the existence of performance trade‐offs between benthic and pelagic habitats. Third, we characterized genetic population structure and then assessed if the degree of divergence in resource use, as a proxy for habitat divergence is associated with the degree of gene flow. Lastly, we assessed the relative effects of resource use and reproductive isolation on body shape variation across populations. Together, we provide a highly integrative analytical approach that allowed us to connect individual‐based and population‐level variation in body shape and size to resource use and genotype under the umbrella of a single study.

## 2 METHODS

### 2.1 Sampling

We sampled 379 adult Arctic charr caught in 2013, 2014, and 2015 from six localities (designated as populations hereafter) in Iceland (Table 1). Throughout the manuscript, we refer to Arctic charr collected from the same lake or stream as a single population, while acknowledging that some populations contain morphs with substantially reduced gene flow. Fish were caught during their spawning periods using gill nets with mesh sizes ranging from 5‐50 mm. We collected fish from four polymorphic populations: Galtaból, Svínavatn, Ϸingvallavatn, and Vatnshlíðarvatn and single morphs from each of Mjóavatn (vatn means lake in Icelandic) and the river population Fljótaá (see Table 1 for sample sizes). Here, we define a morph as a discrete subpopulation that shows some degree of phenotypic, ecological, and genetic differentiation from another subpopulation. For the present analysis, we classify each morph as either benthic or pelagic rather than the more specific descriptors used in previous studies (Table 1). Classification was based on body shape, body size, craniofacial features, and coloration as described previously (Gíslason et al., 1999; Jónsson & Skúlason, 2000; Sandlund et al., 1992; Skúlason et al., 1989, 1999; Skúlason, Snorrason, et al., 1989; Snorrason et al., 1994). We collected a pair of benthic and pelagic morphs from each of Galtaból and Vatnshlíðarvatn and two different benthic morphs and a pelagic morph from Ϸingvallavatn (adequate numbers of a second pelagic morph were unavailable). Fish were photographed on their left side, fork length was measured (from the tip of the snout to the fork of the tail), and white muscle tissue was taken for stable isotope and genetic analyses. Svínavatn has been reported to contain one benthic morph and two pelagic morphs (Gíslason et al., 1999), but based on our analyses, the two pelagic morphs do not differ ecologically or genetically. Thus, we considered the fish from these two groups as a single pelagic morph in all downstream analyses.

**TABLE 1:** Sample sizes of Arctic charr morphs collected from six Icelandic populations and analyzed for variation in stable isotope signatures, morphology, and single nucleotide polymorphisms (SNPs)

Population	Coordinates	Morph	Specific descriptor	Morphology and isotopes	SNPs
Galtaból	Latitude: 65°16ʹ0ʹʹN	Benthic	Small benthic	34	32
	Longitude: 19°43ʹ60ʹʹW	Pelagic	Piscivorous	25	25
Svínavatn	Latitude: 65°12ʹ0ʹʹN Longitude: 20°1ʹ0ʹʹW	Benthic	Large benthic	26	26
		Pelagic 1	Planktivorous	33	32
		Pelagic 2	Piscivorous	33	32
Ϸingvallavatn	Latitude: 64°18ʹ33ʹʹN Longitude: 21°15ʹ00ʹʹW	Benthic 1	Large benthic	33	32
		Benthic 2	Small benthic	33	32
		Pelagic	Planktivorous	33	31
Vatnshlíðarvatn	Latitude: 64°31ʹ0ʹʹN	Benthic	Silver	33	32
	Longitude: 19°37ʹ0ʹʹW	Pelagic	Brown	33	32
Mjóavatn	Latitude: 65°15ʹ34ʹʹN	Benthic	None	31	32
Fljótaá	Longitude: 19°48ʹ31ʹʹW	Pelagic	None	32	32

Note

The more specific descriptors that have been used to name a morph in a given lake in the literature (see text) are also given. The geographic coordinates for each population are shown.

### 2.2 Body shape and size

**FIGURE 1:**
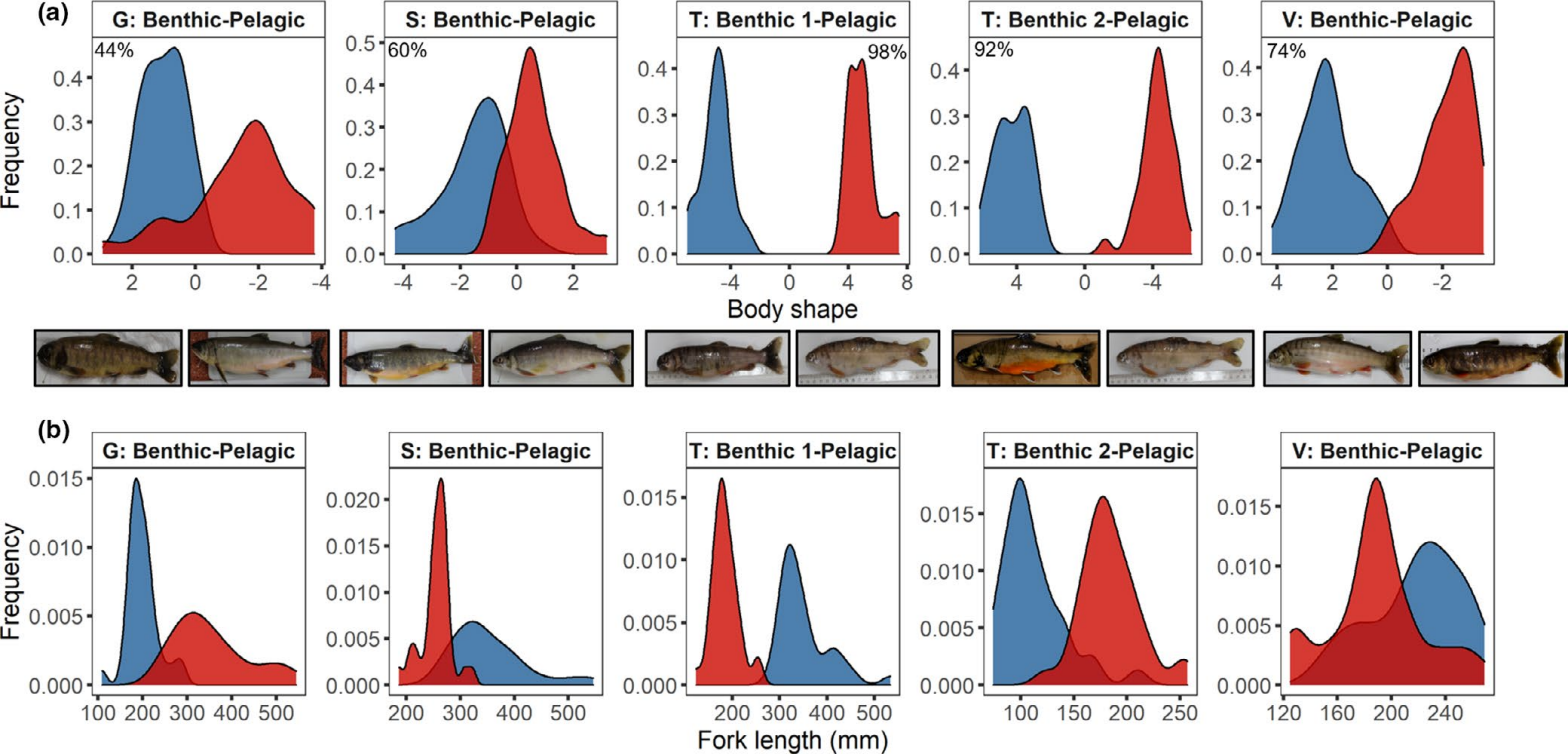
Morphological divergence between benthic and pelagic morphs from four populations of Icelandic Arctic charr. Benthic and pelagic morphs are shown in blue and red, respectively. Populations are denoted as: G—Galtaból; S—Svínavatn; T—Ϸingvallavatn; V—Vatnshlíðarvatn. (a) Body shape variation between benthic and pelagic morphs described by linear discriminant scores, where a linear discriminant analysis was performed on all partial warp and uniform component scores. Percentages denote correct classification of body shape based on being either benthic or pelagic. Images show phenotypic differences in benthic and pelagic morph pairs. (b) Body size variation between benthic and pelagic morphs as measured by fork length

Morphological variation was quantified using landmark‐based geometric morphometrics. The analysis was based on 25 landmarks (Figure 1) placed on individual images (TpsDig (Rohlf, 1990)). Landmarks 1, 22–25 were used to remove variation due to characteristic U‐shaped bending of the dorso‐ventral body axis often observed in dead specimens that is not related to natural body shape variation (TpsUtil (Rohlf, 1990)). Landmarks 23–25 were then removed and not used in further analyses. A generalized Procrustes superimposition (GPA) was performed to remove effects of scale, rotation, and translation for all specimens using Coordgen8, part of the IMP8 software (Sheets, 2014). As is typical in such studies, the effects of allometry (Klingenberg, 2007, 2016) and sex were minimized from the body shape data (see supplementary methods) to focus on phenotypic variation related to habitat and resource use. After minimization of allometric and sex effects, the Procrustes landmark coordinates were input into PCAgen8 (Sheets, 2014) to obtain body shape data based on partial warp and uniform component scores. These scores describe body shape changes localized to a specific region of the body or those occurring uniformly across the body (Zelditch et al., 2004).

### 2.3 Stable isotopes

Carbon (δ^13^C) and nitrogen (δ^15^N) stable isotopic signatures were used as a proxy for resource use (Post, 2002). Organisms that consume benthic prey in northern lakes are expected to have higher δ^13^C isotopic signatures than those that consume pelagic prey. The δ^15^N isotopic signature was used to infer the trophic level of the fish where those from a higher trophic level typically have a higher δ^15^N signature than those at a lower trophic levels. White muscle was dried at 60°C for 48 hr, and samples were homogenized, weighed to 1.25 mg of tissue, and placed into tin capsules. The samples were processed with a continuous flow elemental analysis‐isotope ratio mass spectrometer (Themo Finnigan Delta with Elementar vario ISOTOPE cube). An internationally known internal standard of Mussel tissue (NIST‐2976) was used to standardize carbon and nitrogen measurements to a known reference. δ^13^C values were lipid normalized using the equation: δ^13^C_normalized_ = δ^13^C_untreated_ − 3.32 + 0.99 × C:N, to account for effects of lipids on δ^13^C for aquatic organisms (see Post et al., 2007). Stable isotopic signatures of reference prey baselines were unavailable, so we could not assign morphs to specific resource niches.

### 2.4 Single nucleotide polymorphisms (SNPs)

Characterization of genetic population structure and inferences about the degree of gene flow between pairs of benthic and pelagic morphs were determined from the distribution of single nucleotide polymorphisms (SNPs). Using a phenol–chloroform extraction (Bardakci & Skibinski, 1994; Taggart et al., 1992), DNA was isolated from the white muscle of 371 of the 379 individuals whose morphology was characterized (Table 1). DNA purity was assessed using a NanoDrop^TM^ ND‐8000 spectrophotometer, and samples with high 260/230 readings were treated with a genomic DNA cleanup protocol. DNA concentrations were determined using a Qubit 2.0 Fluorometer and 10 ng/ml samples genotyped with an Arctic charr 87K Affymetrix SNP array (Nugent et al., 2019). The Axiom Analysis Suite (Version 3.1.5.1) was used to process the genotypic data following the best practice workflow specified by Affymetrix. This included specifying a diploid genome, with each individual requiring available genotypes for at least 82% of the SNPs on the array, and a call rate for each SNP greater than 97% across all samples, and the average call rate for individual samples across all SNPs being greater than 98%. A total of 14,187 polymorphic and recommended SNPs were obtained for 369 of 371 genotyped individuals. One fish from Mjóavatn and a pelagic morph individual from Ϸingvallavatn had low‐quality genotypic information and were not used in subsequent analyses.

### 2.5 Data analyses

Data analyses were conducted using the R statistical language (version 3.4.3, R core team, 2017) unless indicated otherwise. Most analyses were only performed with the four polymorphic populations (Galtaból, Svínavatn, Ϸingvallavatn, Vatnshlíðarvatn) and not the two monomorphic populations (Fljótaá and Mjóavatn) as our aim was to assess the relationships among resource use, morphology, and genotype for individual pairs of sympatric morphs. The two groups of pelagic fish in Svínavatn were combined for all analyses as they did not differ in isotopic variation and SNP variation indicating they are not discrete resource‐based morphs. In total, five pairs of sympatric morphs were available for evaluation—one pair in each of Galtaból, Svínavatn, Vatnshlíðarvatn, and two in Ϸingvallavatn.

#### 2.5.1 Morphological divergence

We assessed the degree of morphological differentiation between pairs of sympatric benthic and pelagic morphs with linear discriminant function analyses (LDFA). We used LDFA as it generates an LDFA score for each individual along the primary morphological axis that differentiates a pair of benthic and pelagic morphs. Other approaches generate multiple axes of morphological variation that may not relate directly to the environmental gradient that underlies adaptive divergence in this species. The LDFA axis utilizes all available body shape information, not a subset of variation along separate axes.

For each pair of sympatric morphs, LDFAs were performed on all partial warp and uniform component scores using morph as the grouping variable. Percentages of correct classification into benthic and pelagic morphs were determined using a leave one out cross‐validation. LDFAs were performed using the lda function in the MASS package (Venables & Ripley, 2002). We then tested whether fork length differed among the three morphs in Ϸingvallavatn with one‐way ANOVA (aov function) followed by Tukey's HSD (TukeyHSD function) and among the two morphs in each of Galtaból, Svínavatn, and Vatnshlíðarvatn with two sample *t* tests (t.test function).

#### 2.5.2 Patterns of resource use

Given that niche breadth and variance in resource use should increase as populations diverge into benthic and pelagic morphs from historically monomorphic populations, we assessed whether the four polymorphic and two monomorphic populations differed in the variance of resource use. For this analysis, all fish from a lake population were examined collectively (not subdivided by morph) and their variance in carbon and nitrogen signatures were compared to that of another population using a Levene's test (leveneTest function).

We evaluated within‐population (lake) variation in resource use by determining if δ^13^C and δ^15^N isotopic signatures differed between sympatric benthic and pelagic morphs. We expected that benthic morphs would have stable isotopic signatures associated with the consumption of benthic prey (higher δ^13^C signatures) than fish consuming pelagic prey. We also expected that benthic morphs would have a lower δ^15^N isotopic signature as they typically consume prey from lower trophic levels than pelagic morphs. Given that Ϸingvallavatn has three morphs, a one‐way ANOVA was performed with the aov function:

Isotopic signature ~ Morph

​

Tukey's HSD test was then used to determine which pairs of morphs differed in δ^13^C or δ^15^N isotopic signatures (TukeyHSD function). We tested for significant differences between the pairs of benthic and pelagic morphs in each of Galtaból and Vatnshlíðarvatn with two sample *t* tests (t.test function).

We assessed whether benthic and pelagic morphs from the four polymorphic populations have diverged along similar environmental gradients of resource use using linear mixed effect models. The models were calculated using the lme4 R package (Bates et al., 2015), where morph type (benthic or pelagic) and the alternative isotopic signature were included as fixed effects. The two benthic morphs from Ϸingvallavatn were combined as morphs of the same type should be utilizing similar types of resources, which was confirmed as they did not differ in δ^13^C isotopic signatures (see results). Population was treated as a random effect to account for across population isotopic variance and allow for the generalization of results across populations. The alternative stable isotope signature was included to account for the inherent covariance between the two stable isotopic signatures. The lme4 models were specified as:

δ^13^C ~ Morph type + δ^15^N + (1|Population)
δ^15^N ~ Morph type + δ^13^C + (1|Population)

​

The results for each model were visualized by regressing the isotopic response variable on the fixed isotope factor. The predicted lines of best fit for the models were then overlaid the type of morph.

#### 2.5.3 Predicting body shape and size from resource use

We tested whether resource use was a significant predictor of body shape and size across all individuals within a pair of sympatric benthic and pelagic morphs. Resource use was the predictor variable as it is the presumed agent of selection, while body shape and size, the response variables, are the presumed targets of selection. We determined whether δ^13^C and δ^15^N isotopic signatures predicted body shape (linear discriminant scores from LDFA) and size (fork length) with two sets of linear regression models (lm function). We expected that fish with higher δ^13^C and lower δ^15^N isotopic signatures would have more benthic body shapes (more positive LDFA scores) than those that consume more pelagic prey items. Combining all benthic and pelagic individuals per morph within a single regression analysis allowed us to infer the amount of morphological divergence along the major axis that differentiates sympatric morphs (see Magalhaes et al., 2016). Analyses were performed for each benthic and pelagic morph pair where there were two analyses of shape and two of size for Ϸingvallavatn and one for each of shape and size in Galtaból, Svínavatn, and Vatnshlíðarvatn. The linear regression models for each benthic–pelagic morph pair were:

Body shape ~ δ^13^C + δ^15^N
Body size ~ δ^13^C + δ^15^N

​

The models included both isotope values to take covariation between carbon and nitrogen signatures into account. We tested whether either body shape or size showed linear or nonlinear relationships with isotopic values by comparing AIC values for each model type (Burnham et al., 2011; Murtaugh, 2014). Nonlinear models had the same model structure but were specified as quadratic and cubic by squaring or cubing each fixed factor (poly function). ΔAIC values were calculated to compare the relative strength of each model, where the highest ranked model (lowest AIC score) received a value of zero, and from this, the difference between model‐specific AIC values was calculated. The ΔAIC values were compared for each model using a cutoff value of two to determine model fit (Symonds & Moussalli, 2011). Models with a value greater than two had weaker support than the top‐ranked model and ΔAIC values less than or equal to two were given equal support. If two models had equal support, the least complex model was chosen (e.g., a preference for a linear relationship was given over a nonlinear relationship).

#### 2.5.4 Population genetic structure

We characterized genetic population structure across all populations to determine the degree of genetic differentiation and to infer the amount of gene flow between sympatric morphs. Although studies of Icelandic Arctic charr have detected genetic variation between morphs (Gíslason et al., 1999; Kapralova et al., 2011), we repeated the analyses given that the increased number of genetic markers used here might provide increased resolution of genetic relationships within and among populations. Genetic diversity within and between morphs was estimated by the number of polymorphic SNPs (customs R scripts and PLINK ver. 1.07 (Purcell et al., 2007)), expected (He) and observed (Ho) heterozygosity and nucleotide diversity (π) with Arlequin ver. 3.5.2.2 (Excoffier & Lischer, 2010). We determined the number of distinct genetic groups (*K*) across all populations without a priori morph identification using sparse non‐negative matrix factorization (sNMF) (Frichot et al., 2014) implemented in the R package LEA (Frichot & François, 2015). We tested *K* values from 1 to 15, the number of repetitions was set to 50, and default settings were used for all other parameters to avoid under‐ or over‐estimation of admixture (Rougemont & Bernatchez, 2018). The optimal *K* value was determined by finding the minimum cross‐entropy coefficient (Frichot et al., 2014). We determined whether the identified genetic groups corresponded to morph as identified in the field. We visualized the relationships among genetic groups/morphs with a principal component analysis (PCA), using *pcadapt* (Luu et al., 2017), based on mahalanobis distances and a MAF cutoff of 0.01 followed by the broken‐stick method to confirm the number of genetic groups. PCA was then performed on each polymorphic population separately to determine if population divergence patterns corresponded to benthic and pelagic morph descriptions. Lastly, to further assess genetic relationships among morphs and across populations, we calculated *F*st values between all morphs using 1,000 permutations in *Arlequin* (Excoffier & Lischer, 2010) and constructed the neighbor‐joining tree using an unweighted pair group method (UPGMA) based on Nei's genetic distances followed by a 1,000 bootstrap resampling with the *adegenet* (Jombart, 2008) and *poppr* (Kamvar et al., 2014) packages. The phylogenetic tree was then visualized using *ape* (Paradis et al., 2004) and *ggtree* (Yu et al., 2017).

#### 2.5.5 Associations between resource use and gene flow

We inferred the amount of gene flow between pairs of sympatric benthic and pelagic morphs by determining the proportion of genetic membership that was of benthic or pelagic origin for each individual (ancestry coefficient, q‐value). To achieve this, we conducted sNMF (Frichot et al., 2014) analysis for each of the four polymorphic populations with *K* values ranging from 1 to 5. Individual analyses were performed for each of the two pairs of benthic and pelagic morphs in Ϸingvallavatn. We interpreted that a *q*‐value approaching 1.0 indicated that a high proportion of the genome of benthic ancestry in an individual while *q*‐values approaching 0 indicated that a high proportion of the genome was of pelagic ancestry within an individual. We considered fish with values less than 0.25 and greater than 0.75 to have “pure” morph genotypes and those with intermediate values as “hybrid” genotypes (as denoted in Bittner et al., 2010). A distribution where most q‐values are <0.25 and >0.75 suggests that admixture between a pair of benthic and pelagic morphs is low due to reduced gene flow.

We assessed whether habitat divergence was negatively associated with gene flow across populations. Differences in resource use were used as a proxy of habitat divergence given that benthic and pelagic prey items are located in different areas of the lakes. The estimated percentage of hybrids was used as an indicator of gene flow between morphs. Habitat divergence between each pair of sympatric benthic and pelagic morphs was quantified by calculating ecological distances based on Bhattacharyya distances (Doenz et al., 2019). The Bhattacharyya distances accounted for the covariance of carbon and nitrogen stable isotopic signatures. These are calculated with the Bhattacharyya.dist function within the *fpc* package. Hotelling *t* tests, with 1,000 permutations, were then performed to determine if benthic and pelagic morphs differed in Bhattacharyya (ecological) distances using the hotelling.test function within the *Hotelling* package. A linear model (*lm* function) was used to determine if ecological distance is a significant predictor of the degree of hybridization (hybrid percentage). We expected to see a reduction in the percentage of hybrids between benthic and pelagic morph pairs as the degree of ecological divergence increased.

#### 2.5.6 Relative effects of differential resource use and gene flow on body shape divergence

We assessed if resource use and gene flow have similar or contrasting effects on body shape variation. We regressed all partial warp and uniform component scores against δ^13^C and δ^15^N isotopic signatures using linear regression (lm function) for each benthic–pelagic pair. We performed a similar analysis by regressing ancestry coefficient on body shape. We used all partial warp and uniform component scores to describe body shape variation, instead of LDFA scores, to account for the covariance variance between the different aspects of body shape variation. The LDFA scores also only represented divergence between identified groups, whereas utilizing all partial warp and uniform component scores allowed for a finer scale investigation of body shape variation within each population. The linear regression models implemented were as follows:

Partial warp and uniform component scores ~ δ^13^C
Partial warp and uniform component scores ~ δ^15^N
Partial warp and uniform component scores ~ Ancestry coefficient

​

We then obtained the fitted values of body shape for both sets of regression analyses. The fitted values were the predicted values of partial warp and uniform component scores based on the association to either stable isotope signature or ancestry coefficient. We then determined if the effects of resource use and gene flow on body shape variation were correlated using RV coefficients (Josse & Holmes, 2016), with the coeffRV function in the FactoMineR package (Lê et al., 2008). RV coefficients are an extension of the Pearson correlation for multivariate data and are bounded between zero and one. As we were comparing very large matrices, the probability of detecting a significant result was high, but since the RV coefficient is related to the Pearson correlation (Mukaka, 2012), we could use the size of the coefficient to evaluate the relative effects of resource use and gene flow on body shape (Dunkler et al., 2020; Ho et al., 2019). If the RV coefficients are nonsignificant or significant but close to zero, the fitted body shape values of the two matrices are uncorrelated suggesting that either the magnitude of resource use or genetic divergence has lower relative effects in partitioning body shape variation between benthic and pelagic morphs. If the RV coefficients are significant and approach one, the fitted values of body shape between the two matrices are highly correlated and resource use and gene flow would have similar relative effects in partitioning body shape variation between benthic and pelagic morphs.

## 3 RESULTS

### 3.1 Morphological divergence between benthic and pelagic morphs

Within‐population analyses verified that benthic and pelagic morphs within each of the five morph pairs differed in body shape but to varying degrees based on the percentage of correct classification from the LDFA (Figure 1a; Table S2). The benthic and pelagic morphs in Galtaból showed the greatest degree of overlap in body shape, while the lowest overlap was detected between the benthic 1 and pelagic morphs in Ϸingvallavatn. Morph had a significant effect on fork length in Ϸingvallavatn (*F*_2,96_ = 325.9, *p* < .001), and post hoc testing showed that all three morphs differed significantly (Figure 1b; Table S2). Fork length also differed between benthic and pelagic morphs in Galtaból (*t*_34.4_ = 8.57, *p* < .001), Svínavatn (*t*_28.1_ = 7.0, *p* = <.001), and Vatnshlíðarvatn (*t*_63.7_ = −3.44, *p* = .001).

### 3.2 Patterns of differential resource use

**FIGURE 2:**
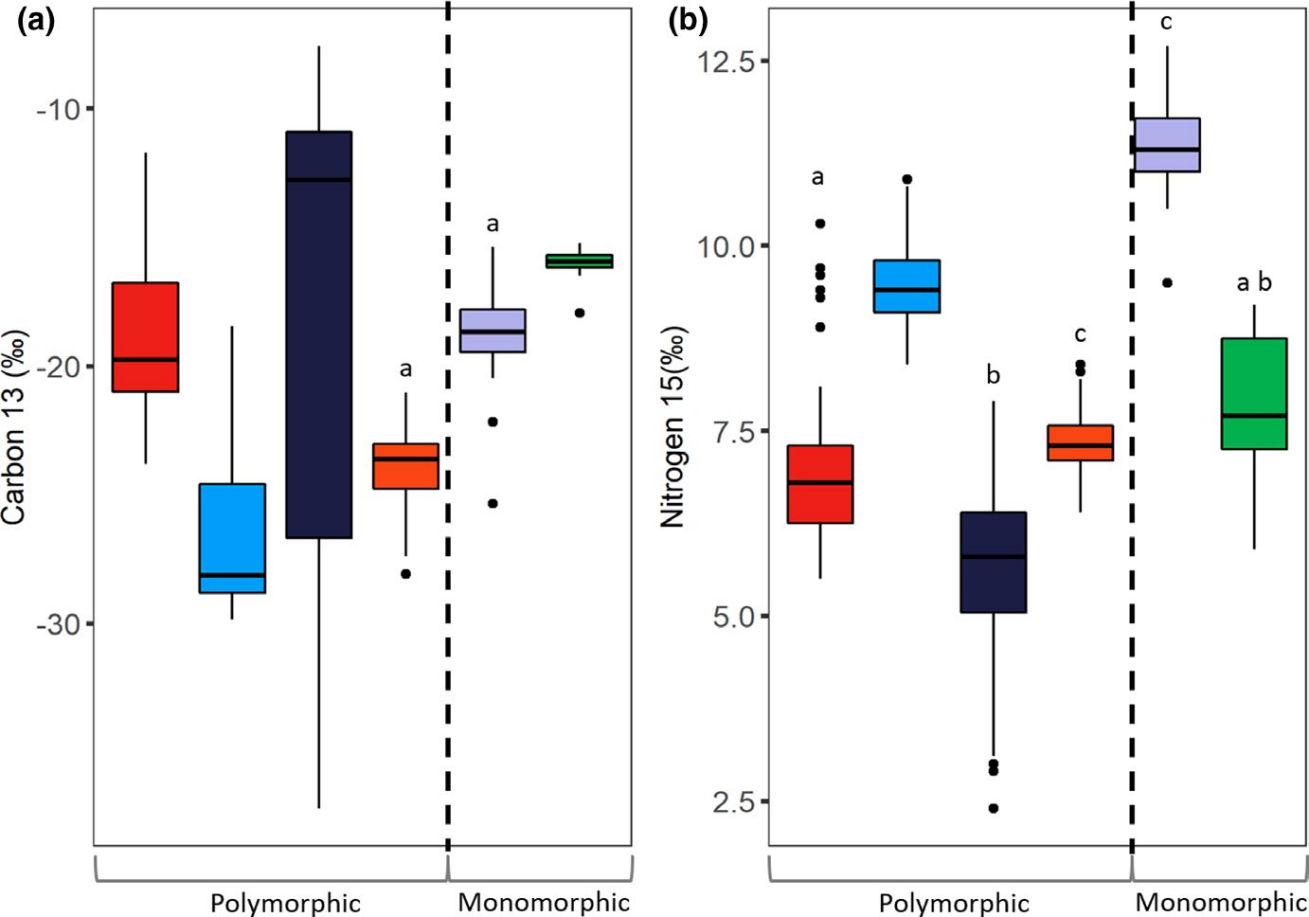
Differences in the variance of carbon (Panel a) and nitrogen (Panel b) stable isotope signatures among four polymorphic (morphs combined) and two monomorphic populations of Icelandic Arctic charr. Populations with similar letters do not differ significantly in variance. Populations in (a and b) shown as: Galtaból—red; Svínavatn –blue; Ϸingvallavatn—purple; Vatnshlíðarvatn—orange; Fljótaá—light purple; Mjóavatn—green

Phenotypic polymorphisms corresponded with a greater range of resource use with three of the four polymorphic populations showing greater variance in δ^13^C isotopic signatures than both of the two monomorphic populations (Figure 2a; Table S2). The variance of the δ^13^C isotopic signatures of Vatnshlíðarvatn (the least phenotypically divergent population) and Fljótaá (monomorphic) did not differ significantly. There were no consistent differences in the variance of δ^15^N isotopic signatures between monomorphic and polymorphic populations (Figure 2b; Table S2).

**FIGURE 3:**
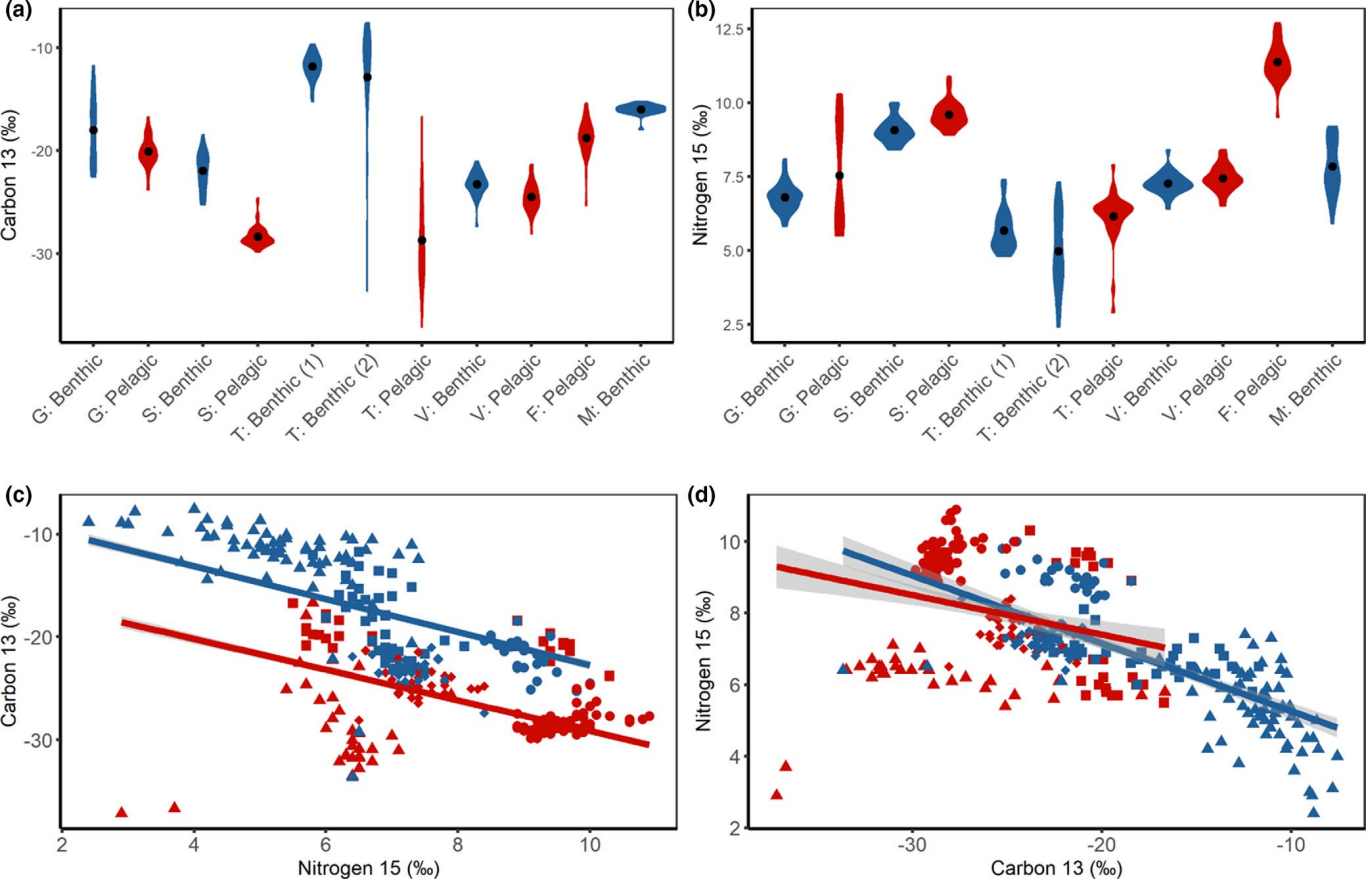
Divergence in δ^13^C and δ^15^N isotopic signatures between benthic and pelagic morphs from six populations of Icelandic Arctic charr. Within all graphs benthic and pelagic morphs are shown in blue and red, respectively. (a) Within‐population comparisons of δ^13^C isotopic signature where black dots represent means. (b) Within‐population comparisons of δ^15^N isotopic signature where black dots represent means. (c) Pattern of divergence in carbon signature between benthic and pelagic morphs across polymorphic populations. The regression lines were fitted using a linear mixed effect model. (d) Pattern of divergence in nitrogen signature between benthic and pelagic morphs across polymorphic populations. The regression lines were fitted using a linear mixed effect model. In panels (c and d), populations are shown by different shapes: Galtaból—square, Svínavatn—circle, Ϸingvallavatn—triangle, Vatnshlíðarvatn—diamond

Within all populations, benthic morphs had significantly higher δ^13^C stable isotope signatures than pelagic morphs as predicted (Figure 3a). Morph type was a significant fixed effect for Ϸingvallavatn (*F*_2,96_ = 153.8, *p* < .001) and post hoc testing indicated that the benthic and pelagic morphs in each pair differed. Similar effects were detected between benthic and pelagic morphs in Galtaból (*t*_1_,_48.1_ = −3.3, *p* < .002), Svínavatn (*t*_1,30.7_ = 17.0, *p* < .001), and Vatnshlíðarvatn (*t*_1,62.5_ = −3.9, *p* < .001). The two benthic morphs in Ϸingvallavatn did not differ in δ^13^C signature.

Differences in δ^15^N signatures between sympatric benthic and pelagic morphs were smaller than those observed for δ^13^C signatures. δ^15^N signatures were significantly higher as predicted in benthic and pelagic morphs in two out of five (40%) comparisons (Figure 3b). Significant effects were found in Ϸingvallavatn (*F*_2,11.4_ = 11.7, *p* < .01) but only between the benthic 2 (small benthic) and pelagic morphs (*p* < .01) and not the benthic 1 (large benthic) and pelagic morphs (*p* = .13). Significant effects were also observed in Svínavatn (*t*_1,46.8_ = −5.4, *p* < .001). However, morphs in Galtaból (*t*_1,26.5_ = 2.0, *p* = .06) and Vatnshlíðarvatn (*t*_1,61.9_ = 1.83, *p* = .07) did not differ statistically but differences were in the same direction (higher δ^15^N signatures in the pelagic morphs as predicted) as observed in Svínavatn and Ϸingvallavatn. The two benthic morphs in Ϸingvallavatn differed significantly in δ^15^N signatures (*t*_1,66_ = −0.70, *p* < .05).

Benthic and pelagic morphs from the four polymorphic populations showed consistent differences in δ^13^C signature based on a linear mixed effect model (Estimate = −6.68, *T*_1,313.5_ = −13.39, *p* < .001) (Figure 3c). The δ^15^N signature fixed effect was also significant (Estimate = 1.33, *T*_88.6_ = −5.40, *p* < .001). The entire model explained 10.0% of the variation in δ^13^C signature. A second model with δ^15^N signature as the response variable indicated no consistent differences between benthic and pelagic morphs across populations (Estimate = 0.20, *T*_1,312.2_ = 1.61, *p* = .11) (Figure 3d). The δ^13^C signature fixed effect was significant (Estimate = −0.05, *T*_313.3_ = −4.67, *p* < .001), and the model explained 6.7% of the variation in δ^15^N signatures across populations.

### 3.3 Predicting morphological variation from resource use

**FIGURE 4:**
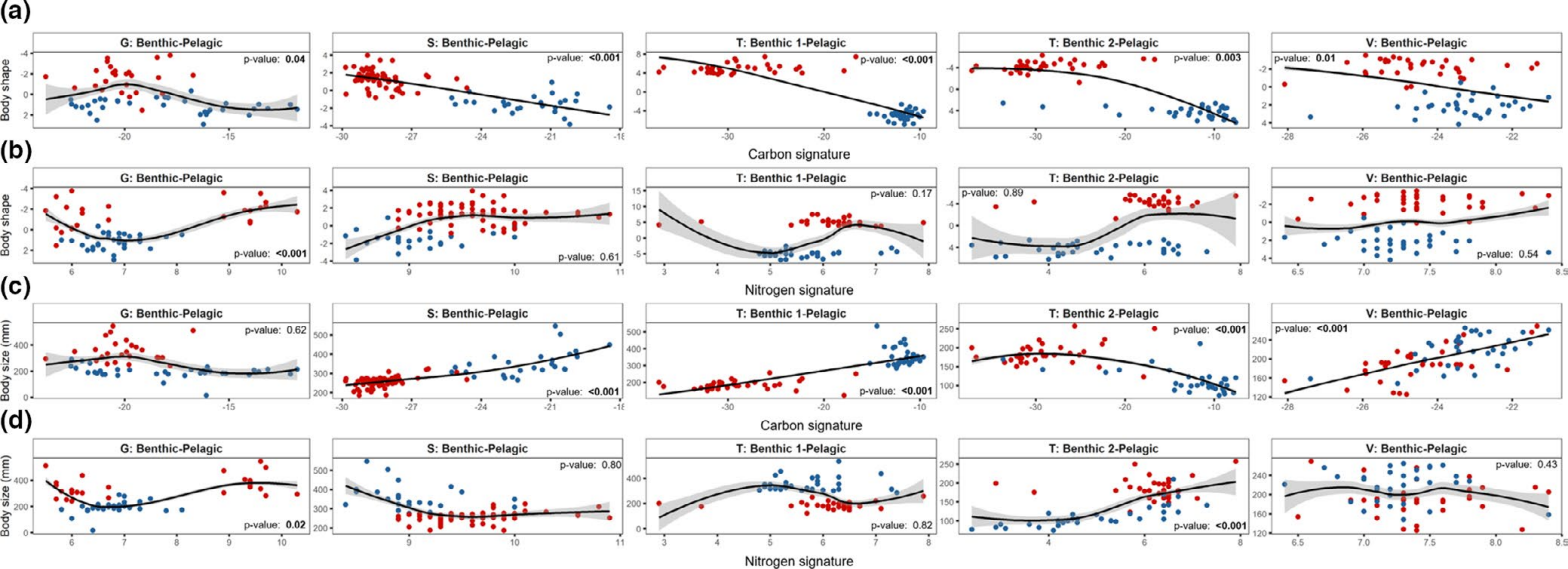
Relationships between morphology (body shape and size) and δ^13^C and δ^15^N isotopic signatures in morphs from four populations of Icelandic Arctic charr. *p*‐values indicate the significance of each relationship. Benthic and pelagic morphs are shown as blue and red, respectively. Populations are denoted as: G—Galtaból; S—Svínavatn; T—Ϸingvallavatn; V—Vatnshlíðarvatn. (a) Relationship between body shape and carbon signature for each benthic–pelagic morph pair. (b) Relationship between body shape and nitrogen signature for each benthic–pelagic morph pair. (c) Relationships between body size and carbon signature for each benthic–pelagic morph pair. (d) Relationships between body size and nitrogen signature for each benthic–pelagic morph pair

Variation in resource use was a significant predictor of body shape matching our prediction. Fish with higher δ^13^C signatures had larger LDFA scores in all five pairs of sympatric benthic and pelagic morphs (Figure 4a; Table S3). The relationship between δ^13^C signature and body size was significant in all lakes except Galtaból (Figure 4c; Table S3). Within Galtaból, there was a significant nonlinear relationship between δ^15^N signature and body shape indicating that the pelagic morph utilized prey from both high and low trophic levels. The relationship between δ^15^N signature and fork length was significant for the morph pair in Galtaból and the benthic 2 (small benthic) and pelagic morph pair in Ϸingvallavatn (Figure 4b,d; Table S3). The best‐supported models for both isotope signatures included linear and nonlinear relationships depending on the response variable (body shape or size) and population (Table S4). The best‐supported linear or nonlinear models for the effects of isotopic signature on body shape or size ranged in their effect sizes (adjusted r‐squared values). Effect sizes for models of isotopic signature predicting body shape ranged from 0.096 in Vatnshlíðarvatn to 0.80 for the benthic 1 (large benthic)—pelagic morph pair in Ϸingvallavatn. The effect sizes for models predicting body size from isotopic signatures were generally smaller but less variable, except in Vatnshlíðarvatn where the effect size was greater than for the models predicting body shape variation (Table S3).

### 3.4 Population genetic structure

For the 369 fish successfully genotyped, 14,187 SNPs were polymorphic in at least one population and 19% of these were polymorphic in all populations based on minor allele frequencies (MAF <0.001; PLINK 1.07 (Purcell et al., 2007)). The number of polymorphic SNPs varied between 4,065 for the pelagic 1 morph in Svínavatn and 1,772 for the pelagic morph in Galtaból (Table 2). Similarly, Svínavatn had the highest number of polymorphic SNPs and Galtaból the lowest. Likewise, morphs and populations varied in levels of genetic diversity and nucleotide diversity, which appears to be lowest in Galtaból and Ϸingvallavatn (Table 2). Fish from Vatnshlíðarvatn and Svínavatn (morphs combined) shared the greatest number of polymorphisms (2,816) while Ϸingvallavatn and Mjóavatn shared the least (1,292).

**TABLE 2:** Overview of genetic diversity in single nucleotide polymorphisms in morphs from four polymorphic populations of Icelandic Arctic charr as estimated by the number of variable SNPs, expected heterozygosity (He), observed heterozygosity (Ho), and nucleotide diversity (π) for each morph and population (morphs combined)

Population	Morph	He	Ho		π	He		Ho		π	Number of SNPs	
		Within morphs				Across morphs					Within morphs	Across morphs
Galtaból	Benthic	0.21	0.22	0.014		0.21	0.16		0.018		3,126	4,473
	Pelagic	0.26	0.28	0.010							1,772	
Svínavatn	Benthic	0.26	0.26	0.019		0.20	0.18		0.024		2,926	6,247
	Pelagic 1	0.24	0.29	0.022							4,065	
	Pelagic 2	0.24	0.25	0.022							3,820	
Ϸingvallavatn	Benthic 1	0.25	0.26	0.013		0.17	0.16		0.015		2,337	4,665
	Benthic 2	0.23	0.23	0.014							2,831	
	Pelagic	0.23	0.23	0.014							2,839	
Vatnshlíðarvatn	Benthic	0.26	0.27	0.020		0.23	0.22		0.021		3,705	5,114
	Pelagic	0.26	0.26	0.021							3,752	
Mjóavatn	Benthic	‐	‐	‐		0.25	0.25		0.019		3,221	3,221
Fljótaá	Pelagic	‐	‐	‐		0.28	0.28		0.025		4,224	4,224

**FIGURE 5:**
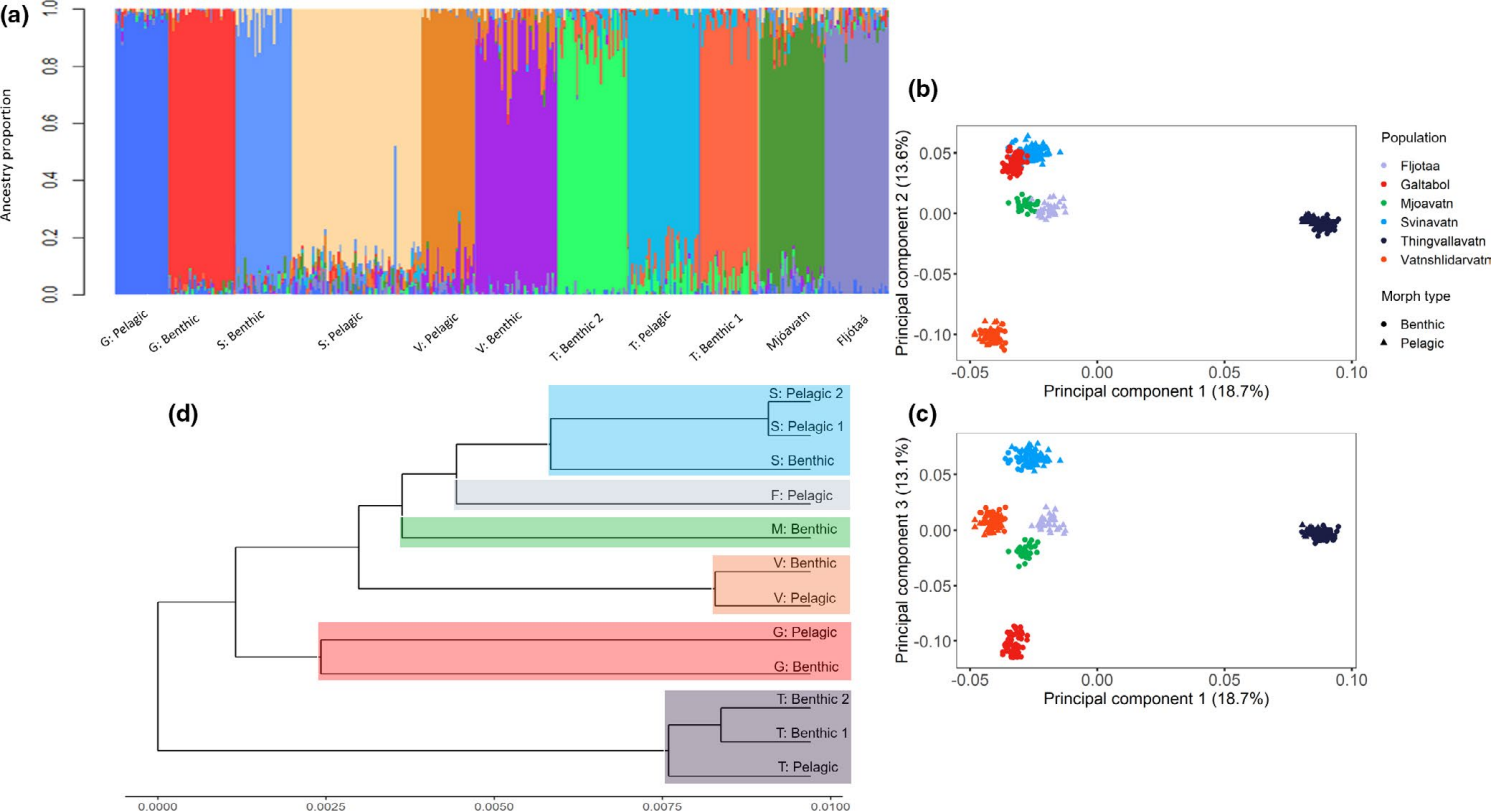
Genetic population structure for six populations of Icelandic Arctic charr ranging from a single morph to up to three morphs. (a) All populations combined (*K* = 11) where each color represents a distinct genetic cluster. Populations are denoted as G—Galtaból, T—Ϸingvallavatn, S—Svínavatn, V—Vatnshlíðarvatn, Mjóavatn, Fljótaá. Morphs are defined as benthic or pelagic. (b) PCA describing genetic variation along PC1 and PC2 for all populations. (c) PCA describing genetic variation along PC1 and PC4 for all populations. Each color represents a distinct population and each shape represents a different morph (circle—benthic, triangle—pelagic). (d) cladogram showing genetic relationships across and within each population. As in the PCA (b & c), each color highlights a distinct population

We detected strong genetic structuring within and among populations. Eleven genetic groupings (*K* = 11) were detected from the sNMF test conducted on all fish from the six populations (Figure 5a; Figure S1). The genetic groups generally corresponded to morphs identified in the field with some mismatches between genetic grouping and morph designation, although the number of mismatches is low within each population. The two pelagic morphs from Svínavatn clustered to the same genetic group and were genetically undifferentiated. The analyses performed within populations returned the same genetic structure (Figures S2‐S4). Similar results were obtained from the PCA as the number of significant principal components was eleven (Figure S5). Ϸingvallavatn separated from the other populations along principal component (PC) 1 (18.7% variance explained), while the other populations were distinguishable along PC 2 and PC 3 (Figure 5b,c). Galtaból and Svínavatn overlapped along PC2 but differed along PC3. PC2 and PC3 explain roughly similar amounts of genetic variation (13.6% and 13.1%, respectively). PCAs performed within each polymorphic population indicated that populations are genetically structured along PC1, which corresponds to divergence of benthic and pelagic morphs (Figure S6). All pairwise Fst values showed significant genetic differentiation among all morphs except for the two pelagic morphs in Svínavatn (Table S6). The neighbor‐joining tree also illustrates the high degree of differentiation among populations and the genetic affinity of sympatric morphs to each other rather than to similar morphs in other populations (Figure 5d).

### 3.5 Genetic admixture and predicting gene flow from divergence in resource use

The majority of fish had ancestry coefficients of either benthic or pelagic ancestry based on the within‐population analyses of genetic structure with sNMF. Most ancestry coefficient values were either <0.25 or >0.75, and only 6.1% of fish had values suggestive of hybrids (Table 3). However, the number of suggested hybrids varied between populations from zero in Galtaból to 15.6% in Vatnshlíðarvatn (2.2% in Svínavatn, 3.2% in Ϸingvallavatn: benthic 1 (large benthic)‐pelagic, and 9.5% in Ϸingvallavatn: benthic 2 (small benthic)‐pelagic). This suggests that gene flow between sympatric morphs varies among populations.

**TABLE 3:** Bhattacharyya ecological distances between benthic and pelagic morph pairs across four polymorphic populations of Icelandic Arctic charr

Population	Morph pair	Ecological distance	Test statistic	*p*‐value	Hybrid percentage (%)
Galtaból	Benthic—pelagic	−0.17	5.14	.012	0
Svínavatn	Benthic—pelagic	5.24	304.18	<.001	2.2
Ϸingvallavatn	Benthic 1—pelagic	37.60	206.67	<.001	3.2
	Benthic 2—pelagic	−10.00	72.84	<.001	9.5
Vatnshlíðarvatn	Benthic—pelagic	0.22	7.74	<.001	15.6

Note

Significance of each ecological distance was determined using a 1,000 permutation Hotelling *t* test.

Benthic and pelagic morph pairs across four polymorphic populations differed significantly in Bhattacharyya ecological distances (Table 3). However, the relationship between ecological distance and the percentage of hybrids across the five pairs of sympatric benthic and pelagic morphs was not significant (*p*‐value = .57; adjusted r‐squared value of −0.17) (Figure S7).

### 3.6 Effects of differential resource use and gene flow on body shape variation

The effects of resource use and gene flow on body shape variation differed across populations (Table 4). The fitted body shape matrices from multiple linear regression analyses with stable isotopic signatures and ancestry proportions were correlated based on the detection of significant RV coefficients in all populations (Table 4). The high RV coefficients for the benthic–pelagic morph pairs in Ϸingvallavatn and Svínavatn indicate a strong correlation between the matrices, which suggests that resource use and genetic divergence are having effects of similar magnitude on body shape variation. In contrast, the RV coefficients were much smaller in Galtaból and Vatnshlíðarvatn indicating a weaker correlation between both matrices, suggesting differences in the effects of resource use and genetic divergence on body shape variation between benthic and pelagic morphs in these populations.

**TABLE 4:** RV coefficients comparing fitted values of body shape (all partial warp and uniform component scores) from multiple regression analyses across polymorphic populations of Icelandic Arctic charr

Population	Morph pair	RV: Carbon‐Nitrogen	*p*‐value	RV: Carbon‐*q*‐value	*p*‐value	RV: Nitrogen‐*q*‐value	*p*‐value
Galtaból	Benthic–Pelagic	0.18	.001	0.12	.008	0.08	.03
Svínavatn	Benthic–Pelagic	0.31	<.001	0.81	<.001	0.24	<.001
Ϸingvallavatn	Benthic 1‐Pelagic	0.07	.03	0.86	<.001	0.12	.005
	Benthic 2‐Pelagic	0.28	<.001	0.73	<.001	0.21	<.001
Vatnshlíðarvatn	Benthic–Pelagic	0.09	.01	0.28	<.001	0.10	.009

Note

Body shape was regressed onto carbon and nitrogen isotopic signatures as well as ancestry proportion.

## 4 DISCUSSION

We assessed several predictions of the hypothesis that ecologically based divergent selection acting on phenotypic variation facilitates adaptive divergence and reduced gene flow between sympatric morphs of Icelandic Arctic charr. Varying degrees of phenotypic, ecological, and genetic variation between sympatric morphs from different populations were observed. However, sympatric morphs showed similar patterns of differential resource use across lakes that appears to be occurring along a common benthic–pelagic environmental gradient. Resource use was a significant predictor of body shape and size suggesting the presence of phenotype–environment correlations, which could reflect the existence of performance trade‐offs in prey capture efficiency. The distribution of single nucleotide polymorphisms between sympatric morphs and populations suggests the independent evolution of morphs within different lakes. Gene flow between sympatric morphs was generally low based on the estimated numbers of hybrids but differs across lakes indicating varied degrees of reproductive isolation. Despite evidence of reduced gene flow between sympatric morphs, we found little support for the prediction that the degree of ecological divergence is associated with the degree of gene flow. However, the comparative analysis of RV coefficients suggests that resource use and reproductive isolation have varied effects on body shape differences between benthic and pelagic morphs across different populations. Our results suggest that relationships among phenotypic, ecological, and genetic divergence vary among populations and may be weaker when reproductive isolation is strong. Thus, reproductive isolation may play a broader role in adaptive sympatric divergence than is often thought (Butlin & Smadja, 2018; Coyne & Orr, 2004).

### 4.1 Differential resource use

Our results suggest that differential resource use has promoted divergence into benthic and pelagic habitats in populations of Icelandic Arctic charr. Polymorphic populations showed greater variation in carbon isotopic signatures compared to monomorphic populations (with few exceptions), suggesting more variable resource breadth by fish in polymorphic populations. These results are similar to studies of a large Arctic charr radiation in Greenland (Doenz et al., 2019). However, our conclusion that monomorphic and polymorphic populations vary in niche breadth remains tentative until the assumption that prey baselines are comparable across populations has been verified.

Sympatric benthic and pelagic morphs from different populations showed similar patterns in δ^13^C isotopic signatures indicating a common pattern of resource use. These results are consistent with previous studies where carbon signatures, as a proxy for benthic and pelagic resource use, are a strong predictor of ecological opportunity for fishes in postglacial lake systems (Doenz et al., 2019; Matthews et al., 2010; Piggott et al., 2018; Siwertsson et al., 2010). However, within‐population differences were smaller for nitrogen signatures as significant effects were detected for only two of the five pairs of sympatric benthic and pelagic morphs. This finding coupled with the lack of a relationship across all populations suggests that variation in trophic position is not a prominent characteristic that differentiates benthic and pelagic morphs in Icelandic Arctic charr. This pattern of resource use divergence has also been seen in Norwegian populations of Arctic charr (Knudsen et al., 2014). Thus, resource use along different trophic positions appears to be more variable than that associated with benthic and pelagic resource use in this species. Such contrasting patterns of resource use among populations can be explained by different properties of local ecosystems that affect niche use. For instance, differentiation in trophic position may require the co‐occurrence of other species, as increasing species diversity has been shown to lengthen the food chain and increase variation in trophic position (Post & Takimoto, 2007; Woods et al., 2018). Icelandic lakes have relatively simple communities, and thus, there are limited opportunities for divergence in trophic position, similar to Arctic charr in Norwegian lakes (Knudsen et al., 2014).

The finding that benthic and pelagic morphs from different populations vary in the degree of differentiation of resource use suggests that local ecological factors may influence their opportunity for adaptive divergence. The difference in carbon isotopic signatures between the morphs in Vatnshlíðarvatn was much smaller than in other populations. This pattern might reflect greater temporal variation in resources compared to other populations due to the shallow nature and small size of the lake with its limited pelagic zone. The benthic and pelagic morphs utilize the same resources when seasonally abundant but the benthic morph switches to alternative resources when the major food source becomes scarce (Jónsson & Skúlason, 2000). Such large pulses of high‐quality prey items may weaken selection for benthic or pelagic resource specialization and morphological divergence (Kristjánsson & Leblanc, 2018). Instead, the benthic and pelagic morphs in Vatnshlíðarvatn appear to have evolved to become resource specialists and generalists, respectively (Franklin et al., 2018; Jónsson & Skúlason, 2000). Other studies have also shown that timing and stability of resource pulses may strongly influence the opportunity and degree to which phenotypic divergence can occur in postglacial lakes (see Landry & Bernatchez, 2010; Landry et al., 2007). As Vatnshlíðarvatn is a small shallow lake relative to the others, divergence along a benthic–pelagic axis may be constrained as smaller lake size has been shown to be correlated with decreases in diversity (Bolnick & Ballare, 2020; Doenz et al., 2019; Lucek et al., 2016; Robinson et al., 2000).

### 4.2 Phenotype–environment correlations

In most populations, resource use was a significant predictor of both body shape and size suggesting that morphs diverging in body shape and size are utilizing different benthic and pelagic resources. The association between benthic and pelagic phenotypes with resource use may arise if the cost of a mismatch (i.e., large effects on survival or reproduction) between phenotype and environment is strong (Camacho & Hendry, 2020). Similar phenotype–environment associations have been observed in other species of northern fishes where body shape, size, armor, and spine traits appear to have arisen in a response to variation in a suite of both biotic (i.e., the presence and density of predators) and abiotic (i.e., ion composition) environmental factors (e.g., Magalhaes et al., 2016). These associations coupled with the finding that phenotype is often related to performance (Arnegard et al., 2014; Berchtold et al., 2015; Ellerby & Gerry, 2011; Schluter, 1995) have provided compelling evidence of performance trade‐offs related to phenotypic variation between habitats. Indeed, a study with Arctic charr from Ϸingvallavatn shows that body shape is related to performance as morphologically intermediated individuals experienced reduced growth rates (Franklin et al., 2018). However, the phenotype–environment correlations reported here are also consistent with other mechanisms such as habitat matching, where individuals disperse to resource patches that match their phenotype and increase their relative performance (Edelaar et al., 2008; Nicolaus & Edelaar, 2018). Another explanation for phenotype–environment correlations is phenotypic plasticity where adult phenotypic variation can be a product of environmental triggers experienced throughout development (Edelaar et al., 2017; Westneat et al., 2019). Phenotypic plasticity appears to interact with genetic effects to determine phenotypic variation in Arctic charr, and that the relative contribution of plasticity can vary between morphs and populations (Adams & Huntingford, 2004; Klemetsen, 2010; Kristjánsson et al., 2018; Parsons et al., ,^2010^, ^2011^). Thus, the phenotype–environment correlations we have observed are most likely affected by the combination of all three factors and the nature of their interplay requires additional studies of their performance and fitness consequences.

### 4.3 Population genetic structure and admixture

Our finding of strong genetic structuring among populations and sympatric morphs is consistent with previous studies of Icelandic Arctic charr (Gíslason et al., 1999; Guðbrandsson et al., 2019; Kapralova et al., 2011). The greater affinity of these morphs to each other compared to similar morphs from other populations supports the hypothesis that morphs are diverging in sympatry independently from other populations. However, we cannot completely exclude the possibility that divergence occurred during a period of allopatry followed by secondary contact as similar contemporary patterns of genetic variation may be generated by alternative divergence histories (Foote, 2018; Ravinet et al., 2017). Indeed, studies of Arctic charr, and other northern fishes, have shown that allopatric divergence followed by a secondary contact event is prevalent (Jacobs et al., 2020; Lehnert et al., 2019; Rougemont & Bernatchez, 2018; Rougeux et al., 2019; Salisbury et al., 2019). Although unlikely due to geography, it is also possible that populations of Arctic charr from lakes not included in this study could have contributed to morph formation. The presumed origin of Icelandic populations from a single glacial refugium (Brunner et al., 2001; Wilson et al., 2004) is inconsistent with the allopatric origin of morphs in different refugia before recolonization of Icelandic lakes. The effects of ecological opportunity on adaptive divergence are a common feature in this species, even in systems where multiple refugia have given rise to contemporary populations (Doenz et al., 2019; Jacobs et al., 2020) suggests that evolutionary history is not the main driver of phenotypic divergence in postglacial systems. Divergence history may have a lesser effect on the evolution of discrete phenotypes if the ecological nature of divergence is common across populations.

Our genetic analysis indicates varied levels of genetic differentiation and admixture between sympatric morphs across lakes. This suggests that gene flow is reduced to some extent in all populations resulting in partial reproductive isolation, a prerequisite for ecological speciation due to ecological opportunity (Langerhans & Riesch, 2013; Wellborn & Langerhans, 2014). Multiple genetic groups were detected in each of the four polymorphic populations through analyses of genetic structure and these groups generally corresponded to morph groups identified a priori (except the two pelagic groups in Svínavatn). However, there were a few individuals in Svínavatn, Ϸingvallavatn, and Vatnshlíðarvatn that showed mismatches between their genotype and phenotype which may indicate potential effects of phenotypic plasticity and/or backcross hybridization (Mandeville et al., 2017). The proportion of hybrids, as inferred from intermediate ancestry coefficients, was low overall but varied across populations. Galtaból showed no evidence of hybridization between the benthic and pelagic morphs, consistent with earlier studies (Gíslason et al., 1999) supporting their status as distinct species (as discussed in Coyne & Orr, 2004). Gene flow between sympatric morphs appears to be occurring in the other three populations with the greatest proportion of hybrids detected in Vatnshlíðarvatn (15.6%). The greater number of hybrids in Vatnshlíðarvatn coupled with the observation that ecological divergence is comparatively low suggests that gene flow may be countering the effects of selection and limiting the completion of reproductive isolation in this population (Hendry et al., 2002; Kraak et al., 2001; Taylor et al., 2006). Gene flow may prevent speciation from occurring if selection is relatively weak and gene flow is small to moderate (Lenormand, 2002; Slatkin, 1987). However, another explanation is that partial reproductive isolation between sympatric morphs may also exist as an evolutionary stable state due to the balance between selection and gene flow (Servedio & Hermisson, 2020). Although our results indicate that gene flow is reduced as shown in other studies (Bittner et al., 2010), the extent to which benthic and pelagic morphs can be considered different species is still up for debate.

### 4.4 Adaptive divergence and gene flow

The relationships among body shape and size, resource use, and gene flow varied among populations at different stages of adaptive divergence. First, habitat divergence, as quantified by ecological distance, was not a significant predictor of gene flow based on the degree of hybridization across populations. Although the expected pattern was observed in three populations, the morphs in the fourth population (Galtaból) appear to be reproductively isolated but this reproductive isolation does not, at least at this time in history, appear to be strongly related to habitat divergence. Second, gene flow and resource use appear to have varied effects on body shape variation across populations. All benthic–pelagic morph pairs (and populations) had significant RV coefficients when relating the effects of carbon and nitrogen signatures to ancestry coefficients, indicating correlated effects of resource use and genetic divergence on body shape variation. However, the RV coefficients were much lower for the morphs in Galtaból and Vatnshlíðarvatn than for those in Ϸingvallavatn and Svínavatn, indicating a weaker relationship between the body shape matrices. The weaker relationship between the body shape matrices can be explained by the finding that differences in resource use between the benthic and pelagic morphs in Galtaból and Vatnshlíðarvatn were much smaller than those in Ϸingvallavatn and Svínavatn; the effects of genetic divergence were relatively high in all populations based on the patterns of genetic structuring and low numbers of hybrids overall. These findings suggest that genetic divergence, and low levels of gene flow, between benthic and pelagic morphs in Galtaból and Vatnshlíðarvatn have a greater impact in driving divergence in body shape than resource use. This implies that resource use, as an agent of selection, might be relatively weak in both Galtaból and Vatnshlíðarvatn while reproductive isolation between the sympatric morphs is stronger and maintains the polymorphism. As the strength of selection often varies temporally in wild populations (Paccard et al., 2018; Siepielski et al., 2009, 2013), reproductive isolating mechanisms may maintain polymorphisms when selection acting on morphology is weak or absent (Butlin et al., 2014; Capblancq et al., 2019; Matessi et al., 2001; Merot et al., 2017; Richter‐Boix et al., 2013; Schluter & Rambaut, 1996). Benthic and pelagic morphs of Icelandic Arctic charr vary in their spawning time and location which has been hypothesized to be due to ecological differences between benthic and pelagic habitats (Sandlund et al., 1992; Skúlason, Snorrason, et al., 1989). However, other reproductive isolating mechanisms, such as behavioral isolation and postzygotic barriers, could also be involved (Kapralova, 2014). The combined effects of divergent selection and reproductive isolation may promote rapid phenotypic divergence in sympatry and maintain divergence through time.

## 5 CONCLUSION

Our highly integrative approach of linking patterns of phenotypic, ecological, and genetic variation has led to increased understanding of the complex interaction of factors that promote adaptive divergence in sympatry. Our results suggest that variation in resource use not only has the potential to facilitate sympatric phenotypic divergence but may also indirectly lead to reductions in gene flow between morphs through habitat divergence. Thus, resource use and habitat divergence may be important in promoting phenotypic divergence and reproductive isolation, but their effects may differ when speciation is complete. Our findings also suggest that reproductive isolation between sympatric morphs can maintain resource polymorphisms even when the agent of selection is comparatively weak. These insights into the evolution of sympatric resource‐based divergence would not have been possible without our approach of connecting variation in phenotype to resource use and gene flow under the umbrella of a single study. We suggest that the use of similar integrative approaches in other organisms will lead to an even greater understanding of the processes that underlie the immense biological diversity in nature.

## CONFLICT OF INTEREST

We declare no conflicts of interest.

## AUTHOR CONTRIBUTIONS

**Matthew K. Brachmann:** Conceptualization (lead); data curation (lead); formal analysis (lead); methodology (lead); visualization (lead); writing‐original draft (lead); writing‐review & editing (lead). **Kevin Parsons:** Conceptualization (equal); writing‐review & editing (equal). **Skúli Skúlason:** Conceptualization (equal); writing‐review & editing (equal). **Moira M. Ferguson:** Conceptualization (equal); funding acquisition (lead); project administration (equal); supervision (lead); writing‐review & editing (equal).

### OPEN RESEARCH BADGES

This article has been awarded Open Data and Open Materials Badges. All materials and data are publicly accessible via the Open Science Framework at https://doi.org/10.5061/dryad.1g1jwstvt and https://github.com/PhDMattyB/Icelandic_Charr_EcoEvo1.

## Supporting information

Supplementary Material

## Data Availability

The data used for all analyses were made publicly available by being deposited on DRYAD (https://doi.org/10.5061/dryad.1g1jwstvt). All R scripts used to generate the analyses are published on GitHub (https://github.com/PhDMattyB/Icelandic_Charr_EcoEvo1).
