# Supplementary material for: The interaction of resource use and gene flow on the phenotypic divergence of benthic and pelagic morphs of Icelandic Arctic charr (Salvelinus alpinus)
Source: Ecol Evol. 2021 May 2;11(12):7315–34. doi: 10.1002/ece3.7563 (PMC8216915; doi:10.1002/ece3.7563)
Supplement: Supplementary file 1 — Supplementary Material [file ECE3-11-7315-s001.docx]

# **Supplementary information**

### *Supplementary methods*

The effects of allometry and sex were minimized from the body shape (partial warp and uniform component scores) for all populations to focus on morphological variation related to ecology. Allometric effects on body shape were minimized within each population using a multivariate linear regression. Partial warp and uniform component scores were regressed onto centroid size (a geometric estimate of body size) using Standardize within Regress8 and residual body shape variation was obtained. The standardization approach is similar to the approach used with single traits however, it must be performed in a multivariate framework due to multiple response variables (affine and non-affine shape components). A linear discriminant function analysis was then performed on the Procrustes landmark coordinates where individuals within each morph were grouped by sex to obtain scores along the axis of morphological sex discrimination. Partial warp and uniform component scores were then regressed against the LDFA scores to obtain sex standardized Procrustes landmark coordinates using Standardize within Regress8. The sex standardized landmark coordinates were then used to calculate new partial warp and uniform component scores using PCAgen8.

### *Supplementary tables and figures*

**Table S1.** Assessing difference in carbon and nitrogen stable isotopic signature variation for six populations of Icelandic Arctic charr. Polymorphic (Galtaból, Svínavatn, Ϸingvallavatn, and Vatnshlíðarvatn) were compared against monomorphic (Mjóavatn and Fljótaá) populations. Stand deviation was calculated for each population (lake) and is denoted by the abbreviation SD

| Population comparison | Isotope | SD lake 1 | SD lake 2 | Variable | Df | F-value | p-value |
| --- | --- | --- | --- | --- | --- | --- | --- |
| Galtaból vs Mjóavatn | Carbon | 2.87 | 0.50 | Lake | 1 | 30.18 | **<0.001** |
|  |  |  |  | Residuals | 88 |  |  |
|  | Nitrogen | 1.26 | 0.65 | Lake | 1 | 0.63 | 0.43 |
|  |  |  |  | Residuals | 88 |  |  |
| Galtaból vs Fljótaá | Carbon | 2.87 | 1.87 | Lake | 1 | 6.47 | **0.01** |
|  |  |  |  | Residuals | 89 |  |  |
|  | Nitrogen | 1.26 | 0.65 | Lake | 1 | 5.42 | **0.02** |
|  |  |  |  | Residuals | 89 |  |  |
| Svínavatn vs Mjóavatn | Carbon | 3.15 | 0.50 | Lake | 1 | 16.09 | **<0.001** |
|  |  |  |  | Residuals | 121 |  |  |
|  | Nitrogen | 0.49 | 0.91 | Lake | 1 | 21.59 | **<0.001** |
|  |  |  |  | Residuals | 121 |  |  |
| Svínavatn vs Fljótaá | Carbon | 3.15 | 1.87 | Lake | 1 | 3.94 | 0.05 |
|  |  |  |  | Residuals | 122 |  |  |
|  | Nitrogen | 0.49 | 0.65 | Lake | 1 | 2.05 | 0.15 |
|  |  |  |  | Residuals | 122 |  |  |
| Ϸingvallavatn vs Mjóavatn | Carbon | 8.90 | 0.50 | Lake | 1 | 25.35 | **<0.001** |
|  |  |  |  | Residuals | 128 |  |  |
|  | Nitrogen | 1.09 | 0.91 | Lake | 1 | 0.57 | 0.45 |
|  |  |  |  | Residuals | 128 |  |  |
| Ϸingvallavatn vs Fljótaá | Carbon | 8.90 | 1.87 | Lake | 1 | 19.04 | **<0.001** |
|  |  |  |  | Residuals | 129 |  |  |
|  | Nitrogen | 1.09 | 0.65 | Lake | 1 | 7.73 | **0.006** |
|  |  |  |  | Residuals | 129 |  |  |
| Vatnshlíðarvatn vs Mjóavatn | Carbon | 1.43 | 0.50 | Lake | 1 | 21.19 | **<0.001** |
|  |  |  |  | Residuals | 95 |  |  |
|  | Nitrogen | 0.40 | 0.91 | Lake | 1 | 29.96 | **<0.001** |
|  |  |  |  | Residuals | 95 |  |  |
| Vatnshlíðarvatn vs Fljótaá | Carbon | 1.43 | 1.87 | Lake | 1 | 0.60 | 0.44 |
|  |  |  |  | Residuals | 96 |  |  |
|  | Nitrogen | 0.40 | 0.65 | Lake | 1 | 6.60 | **0.01** |
|  |  |  |  | Residuals | 96 |  |  |

**Table S2.** Mean differences in body size (fork length) for benthic and pelagic morph pairs across four polymorphic populations of Icelandic Arctic charr. Body size is significantly different between benthic and pelagic morphs within all populations.

| Population | Morph pair | Mean morph 1 | Mean morph 2 |
| --- | --- | --- | --- |
| Galtaból | B-P | 194.1 | 353.9 |
| Svínavatn | B-P | 352.7 | 255.9 |
| Ϸingvallavatn | B1-P | 351.0 | 184.6 |
| Ϸingvallavatn | B2-P | 112.0 | 184.6 |
| Vatnshlíðarvatn | B-P | 218.5 | 189.6 |

**†**Morphs are represented as: B – Benthic & P – Pelagic

**Table S3.** Relationships between morphology (body shape and size) with resource use (carbon and nitrogen stable isotope signatures) for benthic and pelagic morph pairs across four populations of Icelandic Arctic charr. Body shape and size is predicted by carbon signatures across multiple benthic and pelagic morph pairs while, nitrogen signature does not have a strong relationship to body shape size (except in Galtaból).

| Population | Morph pair | Response variable | Fixed factor | Relationship | Estimate | Std. Error | t – value | p-value | Degrees of freedom | F value | Adjusted r-squared value | P-value |
| --- | --- | --- | --- | --- | --- | --- | --- | --- | --- | --- | --- | --- |
| Galtaból | B-P | Body shape | Carbon | Non-linear | -2.9 | 1.4 | -2.1 | **0.04** | 6, 52 | 11.1 | 0.51 | **<0.001** |
|  |  | Body shape | Nitrogen | Non-linear | 0.7 | 1.4 | 0.5 | 0.64 |  |  |  |  |
|  |  | Body size | Carbon | Non-linear | 85.0 | 86.2 | 0.9 | 0.32 | 6, 52 | 10.1 | 0.48 | **<0.001** |
|  |  | Body size | Nitrogen | Non-linear | -225.2 | 90.1 | -2.5 | **0.02** |  |  |  |  |
| Svínavatn | B-P | Body shape | Carbon | Linear | -0.39 | 0.04 | -10.16 | **<0.001** | 2, 89 | 69.0 | 0.60 | **<0.001** |
|  |  | Body shape | Nitrogen | Linear | 0.13 | 0.25 | 0.51 | 0.61 |  |  |  |  |
|  |  | Body size | Carbon | Linear | 15.58 | 1.40 | 11.14 | **<0.001** | 2, 89 | 77.39 | 0.63 | **<0.001** |
|  |  | Body size | Nitrogen | Linear | 2.29 | 8.98 | 0.26 | 0.80 |  |  |  |  |
| Ϸingvallavatn | B1-P | Body shape | Carbon | Linear | -0.5 | 0.03 | -15.3 | **<0.001** | 2, 63 | 131.3 | 0.80 | **<0.001** |
|  |  | Body shape | Nitrogen | Linear | 0.5 | 0.4 | 1.4 | 0.2 |  |  |  |  |
|  |  | Body size | Carbon | Linear | 8.5 | 0.7 | 11.6 | **<0.001** | 2, 63 | 72.3 | 0.69 | **<0.001** |
|  |  | Body size | Nitrogen | Linear | -1.9 | 8.3 | -0.2 | 0.8 |  |  |  |  |
| Ϸingvallavatn | B2-P | Body shape | Carbon | Non-linear | 9.2 | 3.0 | 3.0 | **0.003** | 4, 61 | 39.9 | 0.71 | **<0.001** |
|  |  | Body shape | Nitrogen | Non-linear | 0.3 | 2.5 | 0.1 | 0.9 |  |  |  |  |
|  |  | Body size | Carbon | Non-linear | -65.8 | 35.8 | -1.8 | 0.07 | 4, 61 | 27.9 | 0.62 | **<0.001** |
|  |  | Body size | Nitrogen | Non-linear | 35.7 | 29.4 | 1.2 | 0.2 |  |  |  |  |
| Vatnshlíðarvatn | B-P | Body shape | Carbon | Linear | 0.5 | 0.2 | 2.6 | 0.01 | 2, 63 | 4.5 | 0.10 | **0.02** |
|  |  | Body shape | Nitrogen | Linear | -0.5 | 0.7 | -0.6 | 0.54 |  |  |  |  |
|  |  | Body size | Carbon | Linear | 17.6 | 2.6 | 6.9 | **<0.001** | 2, 63 | 24.62 | 0.42 | **<0.001** |
|  |  | Body size | Nitrogen | Linear | 7.2 | 9.1 | 0.8 | 0.4 |  |  |  |  |

**†** Morphs are represented as: B – Benthic & P – Pelagic

**Table S4.** AIC comparison of linear and non-linear models to determine the relationship between morphology and stable isotope ratios for six benthic-pelagic morph pairs across four polymorphic populations of Icelandic Arctic charr.

| Population | Benthic-pelagic pair | Morphology | Model | AIC | ΔAIC |
| --- | --- | --- | --- | --- | --- |
| Galtaból | B-P | Body shape | Linear | 222.08 | 27.19 |
|  |  |  | Quadratic | 198.91 | 4.01 |
|  |  |  | Cubic | 194.89 | 0 |
|  |  | Body size | Linear | 707.70 | 14.57 |
|  |  |  | Quadratic | 692.54 | 9.41 |
|  |  |  | Cubic | 683.13 | 0 |
| Svínavatn | B-P | Body shape | Linear | 271.33 | 1.72 |
|  |  |  | Quadratic | 269.61 | 0 |
|  |  |  | Cubic | 272.26 | 2.65 |
|  |  | Body size | Linear | 931.73 | 1.56 |
|  |  |  | Quadratic | 930.17 | 0 |
|  |  |  | Cubic | 932.74 | 2.57 |
| Ϸingvallavatn | B1-P | Body shape | Linear | 300.72 | 0 |
|  |  |  | Quadratic | 318.78 | 18.06 |
|  |  |  | Cubic | 335.35 | 34.63 |
|  |  | Body size | Linear | 715.37 | 0 |
|  |  |  | Quadratic | 723.31 | 7.94 |
|  |  |  | Cubic | 733.03 | 17.66 |
|  | B2-P | Body shape | Linear | 315.61 | 5.79 |
|  |  |  | Quadratic | 309.82 | 0.87 |
|  |  |  | Cubic | 308.95 | 0 |
|  |  | Body size | Linear | 636.32 | 0.38 |
|  |  |  | Quadratic | 635.94 | 0 |
|  |  |  | Cubic | 639.47 | 3.53 |
| Vatnshlíðarvatn | B-P | Body shape | Linear | 300.09 | 0 |
|  |  |  | Quadratic | 300.24 | 0.15 |
|  |  |  | Cubic | 300.46 | 0.37 |
|  |  | Body size | Linear | 632.22 | 0 |
|  |  |  | Quadratic | 632.66 | 0.44 |
|  |  |  | Cubic | 633.31 | 1.09 |

**†** Morphs are represented as: B – Benthic & P – Pelagic

**Table S5**. Relationships between ancestry proportion and resource use (carbon and nitrogen stable isotope signatures) between benthic and pelagic morphs within populations of Icelandic Arctic charr. We show differences between ancestry proportion and carbon and nitrogen signatures, as well as the significance of the overall model. Model: Ancestry proportion ~ Carbon 13 + Nitrogen 15.

| Population | Morph pair | Fixed factor | Estimate | Std.Error | t-value | p-value | Null deviance | Residual deviance |
| --- | --- | --- | --- | --- | --- | --- | --- | --- |
| Galtaból | B-P | Carbon | 0.22 | 0.12 | 1.89 | 0.06 | 70.39 | 63.32 |
|  |  | Nitrogen | -0.25 | 0.24 | -1.06 | 0.29 |  |  |
| Svínavatn | B-P | Carbon | 0.92 | 0.09 | 10.24 | **<0.001** | 85.84 | 15.80 |
|  |  | Nitrogen | -0.04 | 0.44 | -0.09 | 0.92 |  |  |
| Ϸingvallavatn | B1-P | Carbon | 0.30 | 0.03 | 10.62 | **<0.001** | 56.91 | 7.52 |
|  |  | Nitrogen | -0.20 | 0.27 | -0.73 | 0.47 |  |  |
| Ϸingvallavatn | B2-P | Carbon | 0.27 | 0.05 | 5.96 | **<0.001** | 57.80 | 15.93 |
|  |  | Nitrogen | 0.29 | 0.26 | 1.12 | 0.27 |  |  |
| Vatnshlíðarvatn | B-P | Carbon | 0.68 | 0.22 | 3.13 | **0.003** | 54.61 | 39.57 |
|  |  | Nitrogen | -1.03 | 0.76 | -1.35 | 0.18 |  |  |

**†** Morphs are represented as: B – Benthic & P – Pelagic

**Table S6.** Pairwise Fst values among all benthic and pelagic morphs from six lake populations of Icelandic Arctic charr. Fst values and associated p-values are shown below and above the diagonal line of 0’s. All Fst values are significant except between the two pelagic morphs from Svínavatn.

|  | **G:Benthic** | **G:Pelagic** | **S:Benthic** | **S:Pelagic1** | **S:Pelagic2** | **T:Benthic1** | **T:Benthic2** | **T:Pelagic** | **V:Benthic** | **V:Pelagic** | **Mjóavatn** | **Fljótaá** |
| --- | --- | --- | --- | --- | --- | --- | --- | --- | --- | --- | --- | --- |
| **G:Benthic** | 0.000 | **<0.001** | **<0.001** | **<0.001** | **<0.001** | **<0.001** | **<0.001** | **<0.001** | **<0.001** | **<0.001** | **<0.001** | **<0.001** |
| **G:Pelagic** | 0.43 | 0.000 | **<0.001** | **<0.001** | **<0.001** | **<0.001** | **<0.001** | **<0.001** | **<0.001** | **<0.001** | **<0.001** | **<0.001** |
| **S:Benthic** | 0.41 | 0.45 | 0.000 | **<0.001** | **<0.001** | **<0.001** | **<0.001** | **<0.001** | **<0.001** | **<0.001** | **<0.001** | **<0.001** |
| **S:Pelagic1** | 0.36 | 0.39 | 0.18 | 0.000 | 0.13 | **<0.001** | **<0.001** | **<0.001** | **<0.001** | **<0.001** | **<0.001** | **<0.001** |
| **S:Pelagic2** | 0.35 | 0.39 | 0.16 | 0.0007 | 0.000 | **<0.001** | **<0.001** | **<0.001** | **<0.001** | **<0.001** | **<0.001** | **<0.001** |
| **T:Benthic1** | 0.54 | 0.59 | 0.46 | 0.40 | 0.39 | 0.000 | **<0.001** | **<0.001** | **<0.001** | **<0.001** | **<0.001** | **<0.001** |
| **T:Benthic2** | 0.51 | 0.56 | 0.44 | 0.38 | 0.37 | 0.08 | 0.000 | **<0.001** | **<0.001** | **<0.001** | **<0.001** | **<0.001** |
| **T:Pelagic** | 0.51 | 0.57 | 0.44 | 0.38 | 0.37 | 0.15 | 0.13 | 0.000 | **<0.001** | **<0.001** | **<0.001** | **<0.001** |
| **V:Benthic** | 0.40 | 0.45 | 0.33 | 0.28 | 0.28 | 0.46 | 0.44 | 0.44 | 0.000 | **<0.001** | **<0.001** | **<0.001** |
| **V:Pelagic** | 0.38 | 0.43 | 0.32 | 0.27 | 0.27 | 0.45 | 0.43 | 0.43 | 0.05 | 0.000 | **<0.001** | **<0.001** |
| **Mjóavatn** | 0.36 | 0.40 | 0.30 | 0.26 | 0.26 | 0.44 | 0.43 | 0.43 | 0.29 | 0.28 | 0.000 |  |
| **Fljótaá** | 0.31 | 0.35 | 0.22 | 0.19 | 0.19 | 0.35 | 0.33 | 0.33 | 0.23 | 0.23 | 0.21 | 0.000 |


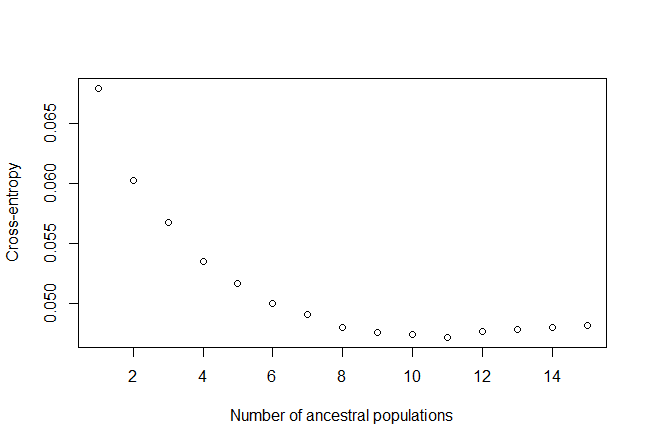


**Figure S1.** Results of a population structure analysis to determine the number of genetically distinct clusters for 12 morphs from 6 populations of Icelandic Arctic charr. The smallest cross entropy value delineates the optimal K value (K = 11).


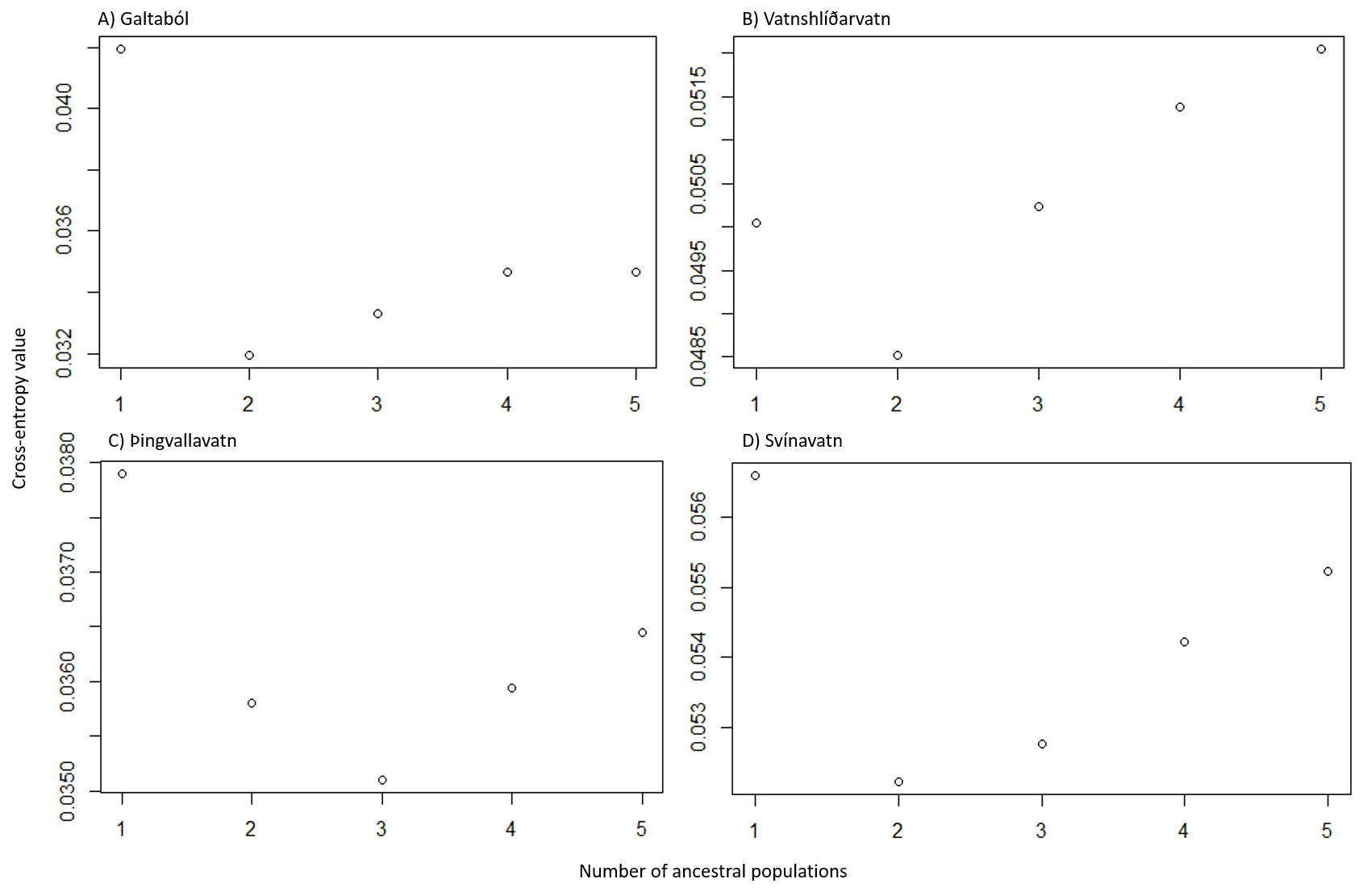


**Figure S2.** Results of a population structure analysis to determine the number of genetically distinct clusters for four polymorphic populations of Icelandic Arctic charr. The smallest cross entropy value delineates the optimal K value for each population. A) Galtaból K = 2. B) Vatnshlíðarvatn K = 2. C) Ϸingvallavatn K = 3. D) Svínavatn K = 2.


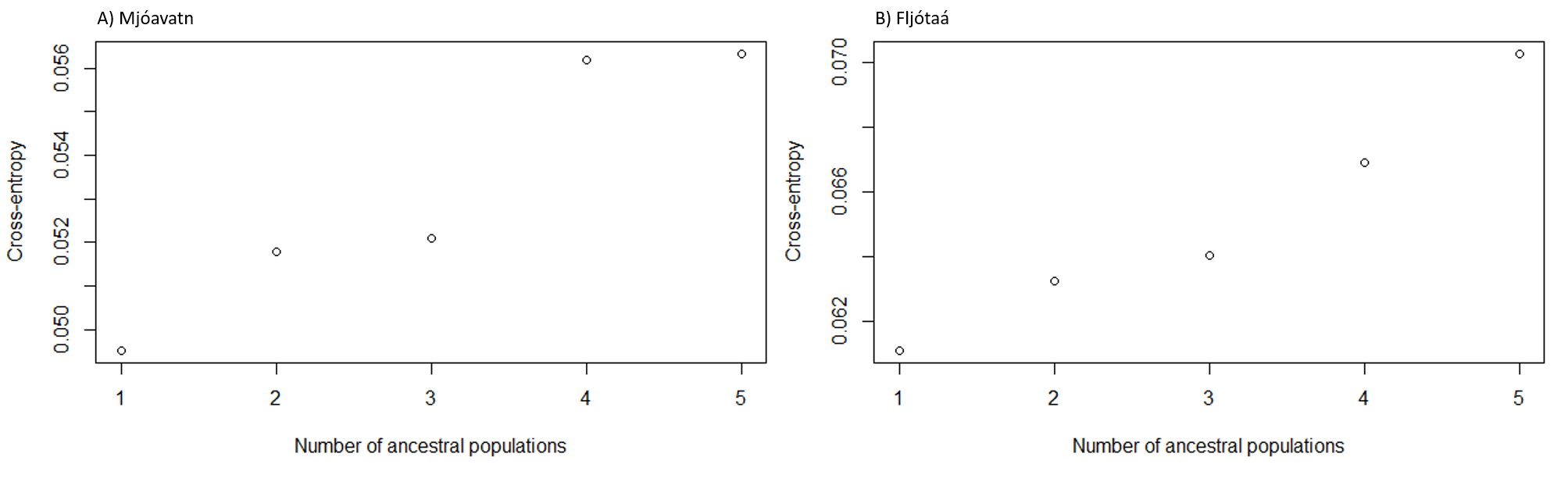


**Figure S3.** Results of a population structure analysis to determine the number of genetically distinct clusters for Mjóavatn and Fljótaá, two monomorphic populations. The smallest cross entropy value delineates the optimal K value for each population. A) Mjóavatn K = 1. B) Fljótaá K = 1.


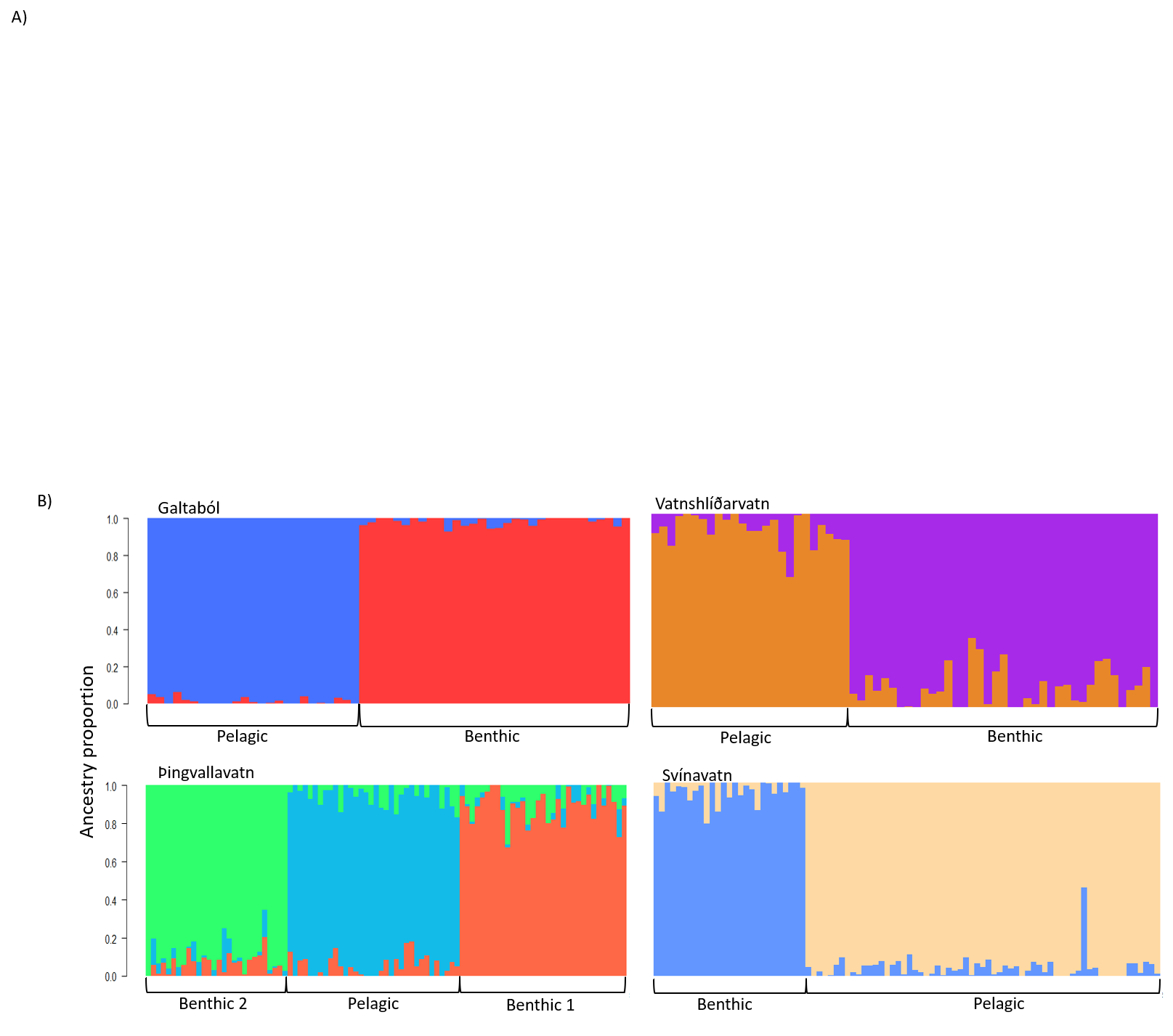


**Figure S4.** Results of a population structure analysis to determine the number of genetically distinct clusters and ancestry proportions within each polymorphic population. sNMF was performed for each population individually.

**
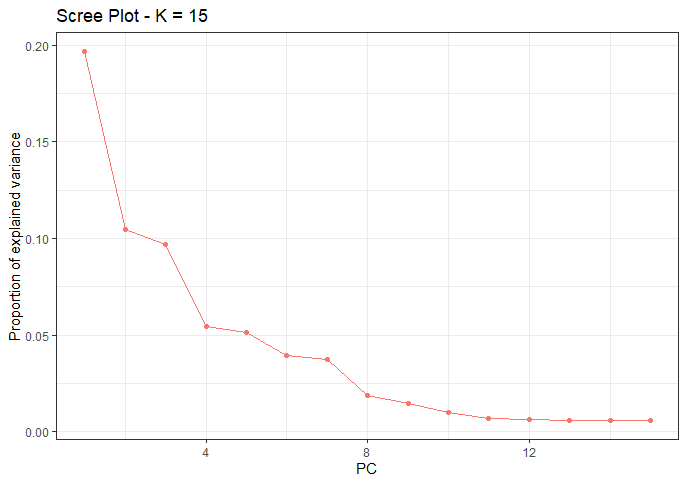
**

**Figure S5.** Results of a PCA performed on all populations of Icelandic Arctic char to assess the number of significant principal components. The number of significant principal components is nine shown by the broken stick visualization.

**
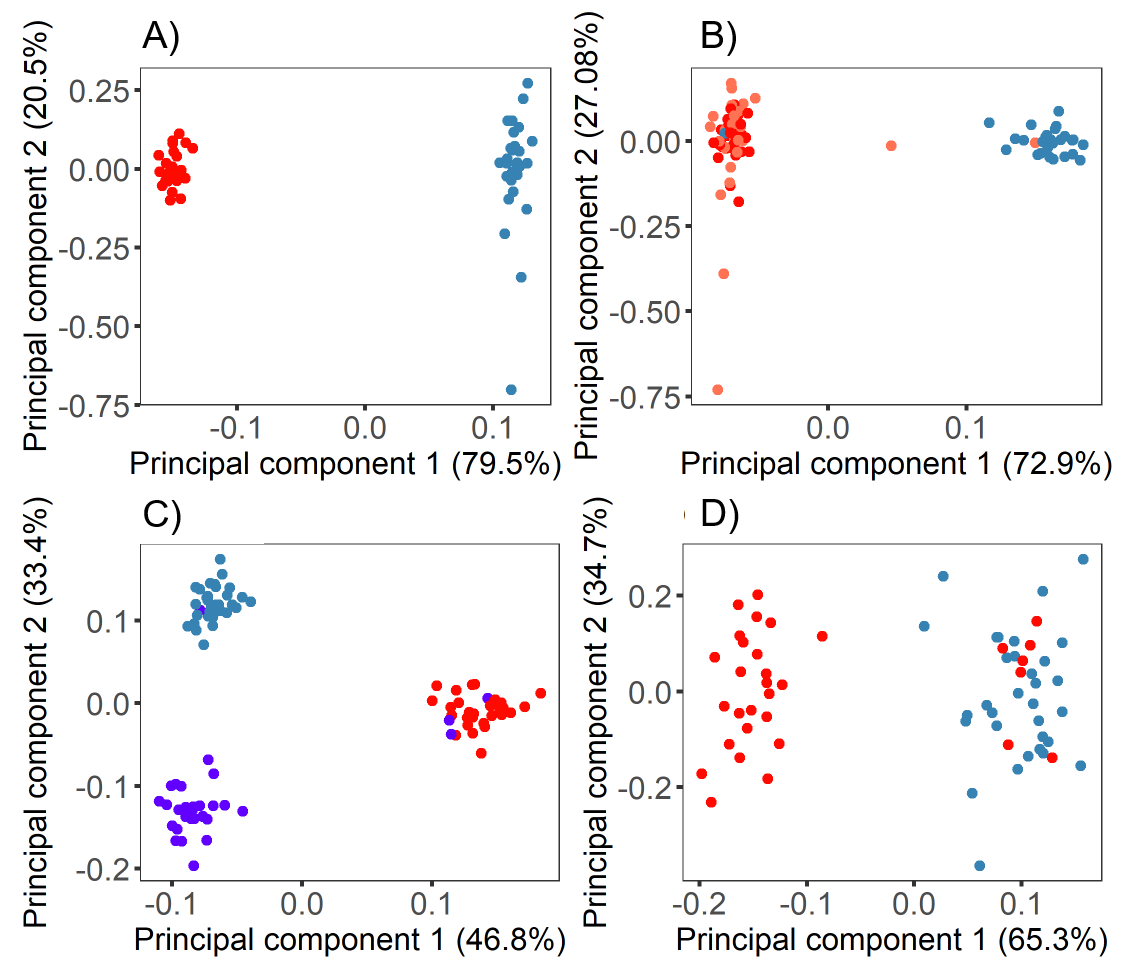
**

**Figure S6.** Results of principal component analyses performed for each polymorphic population individually to determine if populations diverge genetically along a benthic-pelagic axis. All benthic and pelagic morphs are shown in blue and red, respectively. A) Galtaból. B) Svínavatn. C) Ϸingvallavatn. D) Vatnshlíðarvatn


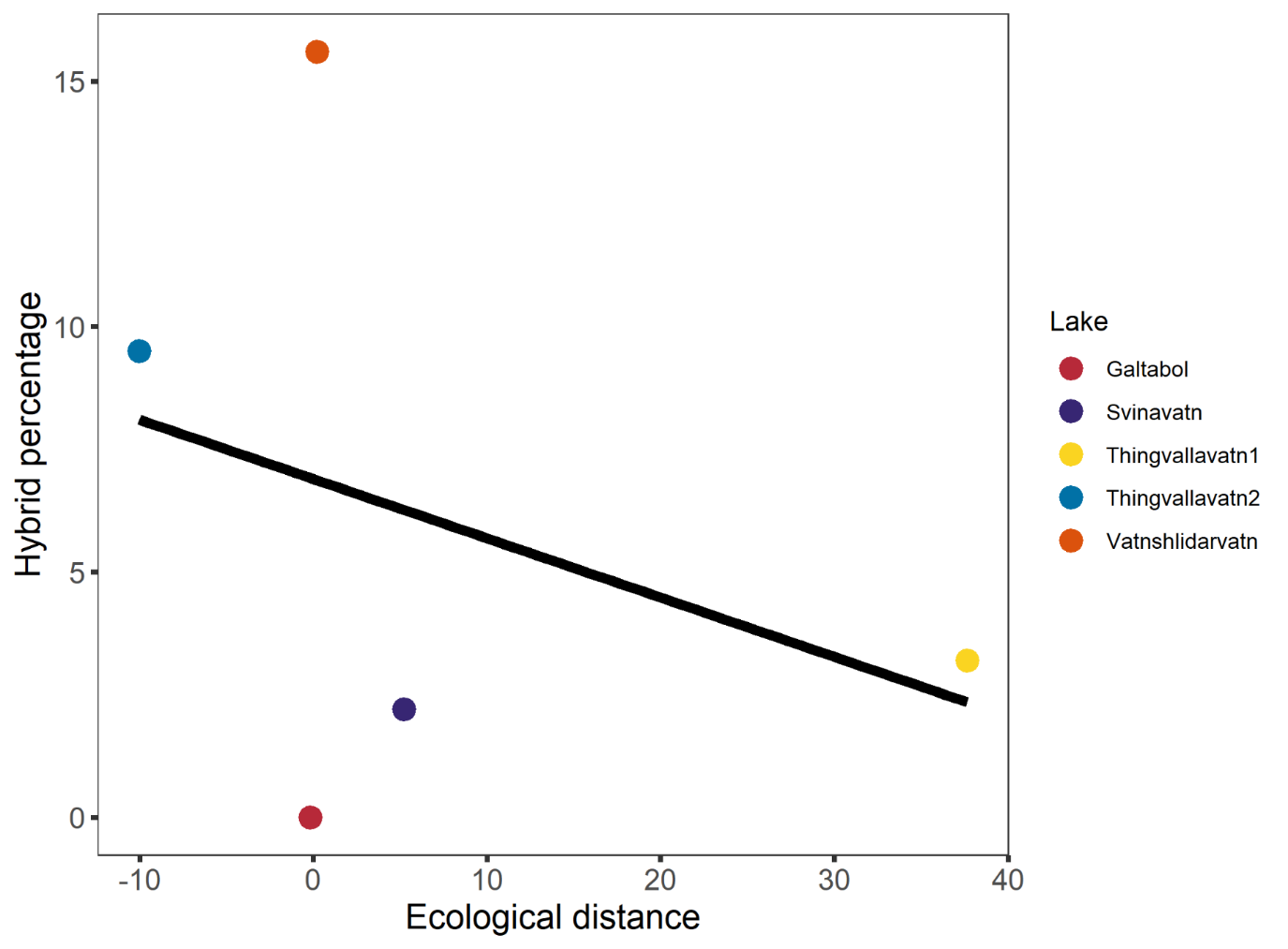


**Figure S7.** Relationship of hybrid percentage and Bhattacharyya ecological distance between sympatric benthic and pelagic morph pairs across four polymorphic populations of Icelandic Arcit charr. Morph pairs are shown in separate colours: Galtaból – Red; Svínavatn – Dark blue; Thingvallavatn benthic 1 – pelagic – Yellow; Thingvallavatn benthic 2 – pelagic – light blue; Vatnshliðarvatn – orange.
